# Capturing RNA Folding Free Energy with Coarse-Grained Molecular Dynamics Simulations

**DOI:** 10.1038/srep45812

**Published:** 2017-04-10

**Authors:** David R. Bell, Sara Y. Cheng, Heber Salazar, Pengyu Ren

**Affiliations:** 1 Department of Biomedical Engineering, University of Texas at Austin, Austin, Texas 78712, United States; 2 Department of Physics, University of Texas at Austin, Austin, Texas 78712, United States

## Abstract

We introduce a coarse-grained RNA model for molecular dynamics simulations, RACER (RnA CoarsE-gRained). RACER achieves accurate native structure prediction for a number of RNAs (average RMSD of 2.93 Å) and the sequence-specific variation of free energy is in excellent agreement with experimentally measured stabilities (R^2^ = 0.93). Using RACER, we identified hydrogen-bonding (or base pairing), base stacking, and electrostatic interactions as essential driving forces for RNA folding. Also, we found that separating pairing vs. stacking interactions allowed RACER to distinguish folded vs. unfolded states. In RACER, base pairing and stacking interactions each provide an approximate stability of 3–4 kcal/mol for an A-form helix. RACER was developed based on PDB structural statistics and experimental thermodynamic data. In contrast with previous work, RACER implements a novel effective vdW potential energy function, which led us to re-parameterize hydrogen bond and electrostatic potential energy functions. Further, RACER is validated and optimized using a simulated annealing protocol to generate potential energy vs. RMSD landscapes. Finally, RACER is tested using extensive equilibrium pulling simulations (0.86 ms total) on eleven RNA sequences (hairpins and duplexes).

## RNA serves important and diverse functions inside the cell

In 1981, Thomas Cech and colleagues observed self-splicing RNA in a 26 S rRNA precursor^1^,^2^. In 1983, Sidney Altman found that ribonuclease P could cleave tRNA in the absence of protein^3^. In 2002, it was discovered that even mRNAs could bind small metabolites and regulate protein expression^4^,^5^,^6^. Today, RNA is recognized as extensively active, with roles in regulating genes, preparatory cleavage, metabolite sensing, and immune response. RNAs achieve this diverse activity through intricately regulated structure, with catalytic RNAs such as riboswitches maintaining highly conserved functional regions^7^,^8^.

## RNA chemistry and the need for accurate structures

RNA structure is a challenge to determine experimentally because it can fold into many different structures. For example, during RNA transcription, synthesized RNA regions fold locally^9^, sampling hairpins and short-range motifs. After transcription completes, RNA molecules are able to fold completely and sample long-range interactions^10^. With the numerous structures available for RNA to fold into, long-lived misfolded RNA intermediates often occur^11^,^12^,^13^. In addition, heterogeneous folding pathways exist for the same RNA sequence^14^,^15^,^16^,^17^,^18^,^19^,^20^. As a result, RNA has a highly dynamic folding landscape, which is challenging to capture using techniques such as x-ray crystallography and NMR spectroscopy^21^,^22^. Further, due to only recent interest in the diversity of RNA function in biology, there is a deficiency in available RNA experimental structures. However, RNA structure is key to understanding its function and for development of RNA-based applications. Due to the lack of available experimental structures of RNA, computational models of RNA are vital to predict RNA structures.

## Secondary structure methods for RNA

Currently, there are a variety of structure prediction methods available to elucidate RNA structure. Secondary structure prediction methods predict base pairing contacts for a given RNA sequence^23^. If homologous sequences exist, comparative sequence analysis^24^,^25^,^26^,^27^ remains the most accurate secondary structure technique. One of the most popular secondary structure prediction methods is dynamic programming. Using nearest neighbor energies^28^ and the sequence of the RNA, dynamic programming methods, such as Mfold^29^,^30^ or ViennaRNA^31^,^32^,^33^, exhaustively compare and build secondary structures to achieve the minimum free energy structure.

However, dynamic programming schemes face certain limitations^34^, such as difficulty predicting pseudoknot structures. Various secondary structure programs^35^,^36^,^37^ have been developed to predict the folding of these structures. Recently, it has been shown that incorporating results from the experimental method SHAPE (selective 2′-hydroxyl acylation analyzed by primer extension)^38^ can moderately increase accuracy of secondary structure prediction^39^,^40^,^41^,^42^,^43^,^44^. Despite its utility, secondary structure prediction is ultimately limited to 2-D base paired RNA structures. For RNA based therapeutics and *de novo* design, 3-D RNA structure must be determined.

## 3-D structure prediction models

Tertiary or 3-D structure prediction methods use template, graph theory, and physics based modeling to sample and predict relevant 3-D RNA structures^45^,^46^. Template based modeling uses predefined, small motifs to assemble RNA structures from their sequence. Template based models include the MC-Fold/MC-Sym pipeline^47^, BARNACLE^48^, RSIM^49^, 3dRNA^50^, RNAComposer^51^, Vfold^52^,^53^,^54^,^55^, RNA-MoIP^56^ and FARNA/FARFAR^57^,^58^,^59^ available in the Rosetta package^60^. Similar to template based modeling, ASSEMBLE^61^ and RNA2D3D^62^ use homologous RNA structures to predict the new RNA structure (with manual refinement available). In graph theory techniques, RNA is depicted topologically to build RNA structures; this improves sampling and even allows for creation of novel RNA motifs. Graph theory techniques^63^ are utilized by RAG/RAGTOP^64^,^65^,^66^,^67^ and others^68^,^69^,^70^,^71^. In physics based methods, the RNA is built from sequence into a 3D structure, and these 3D RNA structures are sampled using Monte Carlo or Molecular Dynamics (MD) protocols. Due to the high charge density of RNA and the associated large computational cost to sample structures, many tertiary structure models use coarse-grained representations of RNA^72^.

In coarse-grained (CG) models, atomic sites are grouped together and represented as a “bead” or pseudoatom. Typical coarse-grained models depict a few pseudoatoms per nucleotide. This results in a reduction in the degrees of freedom and lowers the simulation cost of the model, as compared with simulating the all-atom structure. Physics based coarse-grained models with one pseudoatom per nucleotide include YAMMP/YUP^73^,^74^, an adaptable user input required model, and NAST^75^,^76^, which assumes ideal helices from secondary structure and uses MD and clustering to build loops. iFoldRNA^77^,^78^, Denesyuk *et al*.^79^,^80^, and TOPRNA^81^,^82^,^83^ use three pseudoatoms per nucleotide to depict phosphate, sugar, and nucleobase groups. iFoldRNA uses discrete Molecular Dynamics and replica exchange Molecular Dynamics to sample structures, with non-bonded parameters decomposed from nearest neighbor energies. Similarly, the model by Denesyuk *et al*.^79^,^80^ derives its parameters from nearest neighbor energies and experimentally determined structures. TOPRNA captures effects of secondary structure constraints on loop conformations and free energies. HiRE-RNA^84^,^85^,^86^ depicts six-seven pseudoatoms per nucleotide with five pseudoatoms along the backbone. SimRNA^87^,^88^, Bernauer *et al*.^89^, as well as the previous generation and current RACER model studied^90^,^91^,^92^, all represent RNA with five pseudoatoms per nucleotide. SimRNA uses a Monte Carlo sampling algorithm with parameters from statistical potentials. The model by Bernauer *et al*. similarly uses statistics from high-resolution crystal structures for parameterization yet also derives all-atom potentials for structure refinement.

## The RACER RNA Model

The CG RNA model RACER (RnA CoarsE-gRained) developed and applied in this work is a physics-based model, derived from RNA structural statistics, refined using RNA thermodynamics, and applied in molecular dynamics simulations of folding and complexation of RNAs. In the results section, we first introduce the potential energy functions used in the RACER model, with a focus on the newly implemented effective vdW potential. Second, we demonstrate how RACER parameters were optimized using statistical potentials derived from PDB statistics. Additionally, we provide motivation for modeling RNA as a modeling RNA as a 1D molecule and the associated 1D correction we made to the non-bonded PMFs. Third, we show how we validated RACER using simulated annealing simulations for RACER structure prediction capability and generation of funnel free energy landscapes. Fourth, we apply RACER to generate folding free energy predictions for a testing set of RNA hairpins and duplexes, and we compare our results to experiments. In the discussion section, we summarize the changes made to the RACER model and emphasize RACER’s ability to capture folding free energies and to predict structures. In the methods section, we show (1) the ability of RACER to map between all-atom and coarse-grained representations for use in multiscale simulations, (2) details on the folding free energy calculations, and (3) implementation instructions for those wishing to use RACER.

## Results

### Model

#### Potential energy functions

**Figure 1:**
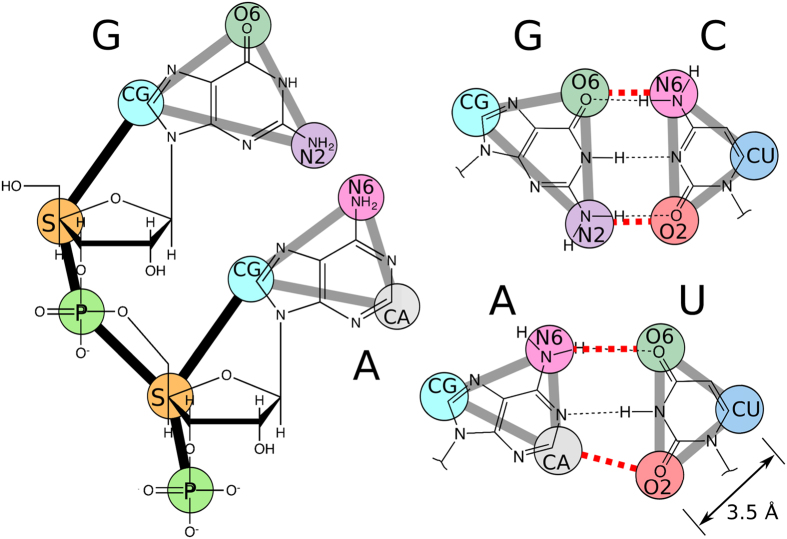
RACER model pseudoatoms overlapping all-atom structure. RACER bonds are shown in bold lines. (Left) nucleobases with backbone. (Right) GC and AU basepairs with hydrogen bonds shown in red dashed lines. Scale bar is shown in lower right.

The total potential energy function of the RACER model includes bond stretching, angle bending, torsion, effective vdW, hydrogen bonding, and electrostatics, labeled as *E*_*bond*_, *E*_*angle*_, *E*_*torsion*_, *E*_*vdW_eff*_, *E*_*hb*_, and *E*_*ele*_ respectively (see Eq. 1). The RACER model is currently implemented in TINKER^93^. In RACER, RNA nucleotides consists of 5 pseudoatoms per nucleotide, with a total of 9 pseudoatom types (shown in Fig. 1). The RACER model used here differs from previous publications^90^,^91^ in that we employ a novel effective vdW potential to better capture the short-range non-bonded interactions among the pseudoatoms, which we found to be essential for correctly capturing the folded state. As a result, we had to re-parameterize the other non-bonded contributors including the electrostatics and hydrogen bonding potential.


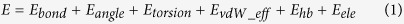


#### Bonded Potential Energies

The potential energy functions which retain the same functional form between the previous model and RACER are the bonded potential energy functions. Bond and angle potentials are represented by harmonic terms: 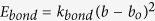 and 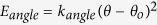. The torsion potential of Eq. 2 uses the first 3 terms of a Fourier series expansion for the torsion potential, where *ϕ* is the torsion angle, and *k*_*n*_ and *δ*_*n*_ are the spring constant and phase angle of expansion term *n*.


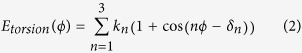


#### Improved Effective vdW Potential

**Figure 2:**
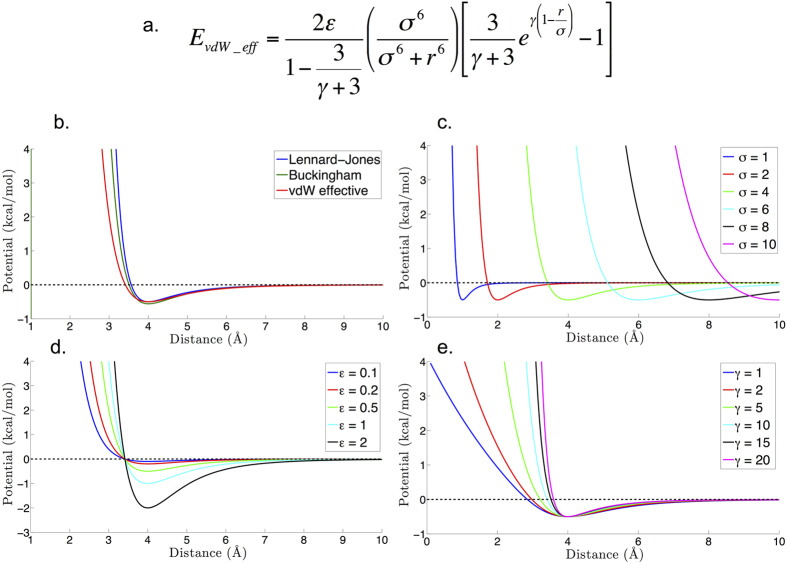
vdW_eff_ potential. (**a**) Functional form. (**b**) Effective potential compared to standard Lennard Jones and Buckingham potentials with minimum energy potential ε = 0.5 kcal/mol, minimum energy distance, σ = 4 Å, and gamma of effective potential γ = 10. (**c**) Effect of changing value of minimum energy distance, σ (**d**) Effect of changing minimum energy potential, ε (**e**) Effect of changing the short range behavior with parameter γ. For (**c**–**e**), unless stated, ε = 0.5 kcal/mol, σ = 4 Å, and γ = 10.

The RACER model includes a newly implemented effective potential (vdW_eff_) that significantly improves the fit of RACER to non-bonded statistical potentials. In the previous model^92^ the vdW-like non-bonded potential was modeled using a Buckingham function. However, this was found to significantly overestimate repulsion at short distances when compared with statistical potentials. The new effective vdW_eff_ potential (Eq. 5) allows for tuning the repulsion at short distances through a third parameter γ, enabling a closer fit to the statistical non-bonded potential of mean force (PMF) (Fig. 2).

The vdW_eff_ does not represent the true vdW interaction, but rather the potential of mean force between a pair of pseudoatoms. However, based on statistical potentials, the non-bonded interactions between most pairs of pseudoatoms we sampled exhibited vdW potential-like behavior. The new functional form for vdW_eff_ potential taken from ref. 94 is shown in Eq. 3, where ε is the minimum well depth and σ is the distance of minimum energy, and γ is a parameter allowing for fine-tuning of the slope of the short-range interaction. Figure 2b presents a comparison between the vdW_eff_, Lennard Jones, and Buckingham potentials while Fig. 2c–e show the effects of the three parameters σ, ε, and γ on the vdW_eff_ potential. The combining rules for unlike pseudoatom types *i* and *j* in the vdW_eff_ potential are: 
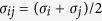
, 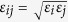, and 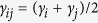.


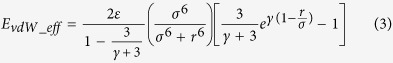


#### Hydrogen Bond and Electrostatics Energies

The hydrogen bond (Eq. 4) and Debye-Huckel electrostatics (Eq. 5) potential energy terms are of the same form as used previously. However, we reparametrized the hydrogen bond and Debye-Huckel potentials with the introduction of the new vdW_eff_ term. In the hydrogen bond potential *ε*_*hb*,*max*_ is the maximum potential found at the hydrogen bond equilibrium distance *σ*_*hb*,*eq*_.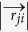 is the magnitude of the vector from atom j to atom i, while 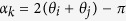 is a directional component with *θ*_*i*_ and *θ*_*j*_ defined in Fig. S1. For hydrogen bond parameterization, the maximum potential *ε*_*hb*,*max*_, was increased from 0.5 kcal/mol to 2.0 kcal/mol. Other hydrogen bond parameters including equilibrium distance *σ*_*hb*,*eq*_ of 2.9 Å and cutoff of 6 Å (base edge) remain the same as the previous model. Hydrogen bond potential energy is computed for both canonical (GC, AU) and noncanonical base pairs. For Debye-Huckel Eq. 5, *q*_*i*_ is the charge of atom i, *r*_*ij*_ is the distance between atom i and atom j, *D* is the dielectric constant, and *ξ* is the Debye length. A dielectric constant *D* of 25 was determined to be optimal under the new model potential, compared to 78 from the previous model. In depth discussion of Debye-Huckel and hydrogen bond optimization can be found in the SI.


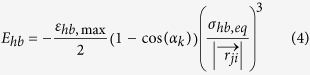



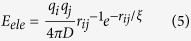


### Model improvement and Parameterization

#### Statistical potentials

The premise of our parameter optimization was to fit to both RNA structure and experimental free energies. First, we updated model statistical potentials from experimentally determined crystal structures. We downloaded all available Protein Data Bank (PDB, http://www.rcsb.org/) RNA structures as of RNA structures as of Feb. 10, 2015, (excluding RNA-protein and RNA-DNA combination structures) totaling ~1100 entries. Our previous model fit to statistical potentials used approximately 668 structures. For RACER, our updated parameterization includes an additional ~400 structures, which led to various modifications in the potentials. The method of statistical potentials involves fitting energy functions to statistically derived potential of mean force (PMF) curves. The PMFs are determined by taking the probability distribution *P*(*r*) of occurrences from the PDB structure set and then extracting the free energy *G*(*r*), 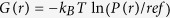 with the reference distribution *ref* setting the minimum interaction at 0 kcal/mol.

One of the major improvements in the current model is to adopt a new nonbonded effective potential form to capture the intricate short range behavior observed in nonbonded statistical potentials that standard vdW potential forms (including our model’s previously used Buckingham potential) cannot capture. The Buckingham and other common vdW functions are too stiff at short range with a steep slope, whereas the nonbonded statistical potentials reveal much softer behavior. We have identified a more “flexible” vdW_eff_ potential that better captures this short range behavior, which is critical for local packing of RNA molecules. When we implemented the new potential, it was also necessary to re-parameterize the torsion, electrostatics, and hydrogen bond interactions for consistency.

#### 1-D PMF for RNA

In this work, we determined that modeling RNA as one-dimensional rather than a three-dimensional, isotropic molecule is more appropriate when extracting the statistical potentials from PDB structures. This choice is justified as there is an abundance of short, linear helices found in PDB structures of RNA. Additionally, folded RNA typically forms prolate ellipsoids^95^. Similarly, in the PDB structure of 16S rRNA more than half of the nucleotides are base paired^24^. Therefore, treating RNA as a one-dimensional molecule for capture of local interactions is not unreasonable. Additionally, 3D PMFs are more appropriate for systems with isotropic distance distributions, such as molecular liquids^96^,^97^,^98^ and proteins^99^,^100^,^101^.

**Figure 3:**
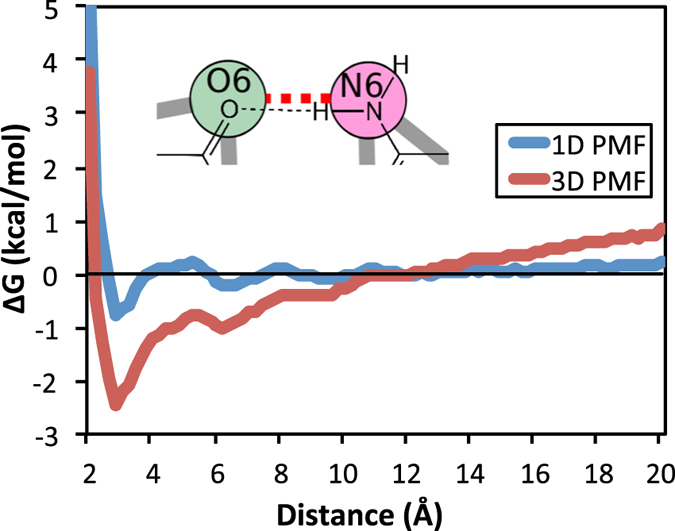
Comparison of a 1-D and 3-D statistical potential PMF, computed from a 1-D and 3-D radial distribution function respectively. Note how the 3-D PMF continues to diverge at long distances whereas the 1-D PMF falls off to zero. This statistical potential is that of RACER base pseudoatoms O6-N6.

Our motivation for modeling RNA as a 1D molecule came from the observation of divergence of 3D radial distribution functions (RDF) at distances greater than 10 Å, and as a result the potential of mean force (PMF) that was derived from the RDF did not converge to zero at large separation (see Fig. 3). The cause of this divergence at long distances is the inherent volumetric effect of the 3D RDF, while the PDB structures we sample are mostly small and linear. The statistical potentials do include some larger ribosomal structures, but these are too few to cause the observed divergence. Contrary to 3D RDFs, when 1D radial distribution functions were used the PMF asymptotically approached zero for long distances (see Fig. 3), reinforcing the discussion that the set of RNAs used here in statistical potentials can be adequately sampled as linear 1D, rather than 3D RDFs. The main difference between 3D and 1D RDFs is the normalization factor. For 3D RDFs, normalization is done over a volumetric shell *4πr*^*2*^*dr*, whereas 1D RDFs normalizes over an incremental distance, *dr*.

Specifically, the non-bonded PMF is evaluated via Boltzmann inversion as 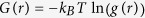 where *g*(*r*) is the radial distribution function, normalized probability function discussed above. When treating RNA as a 3D isotropic molecule, the 3D RDF, as was done previously^90^,^91^, is given by 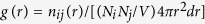, where *n*_*ij*_(*r*) is the number of atom type *j* at distance *r* from atom type *i, N*_*i*_ and *N*_*j*_ are the total number of *i* and *j* atoms respectively, and *V* is the volume of the system. Now we treat RNA as a “1D”, linear molecule to more adequately parameterize the vdW_eff_ potential, and the RDF becomes 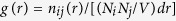.

### Structure Prediction

#### Folding RNA by simulated annealing

We tested RACER with simulated annealing simulations to (1) validate that RACER can accurately fold experimentally determined RNA structures and to (2) ensure the native structure has the lowest energy on its energy landscape. We ran simulated annealing simulations on a testing set of 14 RNAs, duplexes and hairpins, that have known experimentally determined structures^90^. This test set of 14 RNAs was included as part of the 1100 structures used to compute our statistical potentials; however, the contributions of the 14 RNA test set (~1% of training set) to the statistical potentials and thus parameterization is negligible. From annealing simulations on this set of 14 RNAs, RACER is able to predict 13 out of 14 RNA molecules with RMSD < 5 Å, and 6 RNA molecules with RMSD < 2.5 Å. The average RMSD between the predicted lowest-energy structures and native structures is 2.93 Å. This average RMSD is improved from our previously published average RMSD of 3.31 Å; additionally, our model now has the capability to predict free energy landscapes of RNA in addition to structure prediction.

The simulated annealing protocol involved running MD sequentially for 5 ns at temperatures in order of 298(K), 400, 1000, 900, 800, 700, 600, 500, 400, 298 K, for a total simulation time of 50 ns, with structures saved every 10 ps. Given the high temperatures used, we used a 1fs time step for annealing simulations. Results for structure prediction using simulated annealing are given in Table 1. These predicted RMSD values are calculated between PDB structures and the minimum potential energy structures found by RACER.

#### Energy landscapes

Analyzing the energy landscapes of the 14 RNAs in our training set was an important part of our optimization. RNAs are complex molecules that may adopt stable and long lived misfolded structures. However, it is assumed the final native structures, at least *in vitro*, should have the lowest free energy for the given environment^102^. Here, annealing simulations are used to generate a large number of unfolded structures for each RNA. Each of these structures is then energy minimized to 0 K. The energy and RMSD (with respect to the native structure) of each structure are used to characterize the energy landscape. The energy-RMSD landscapes for all 14 RNAs are given in SI, Table S1.

**Figure 4:**
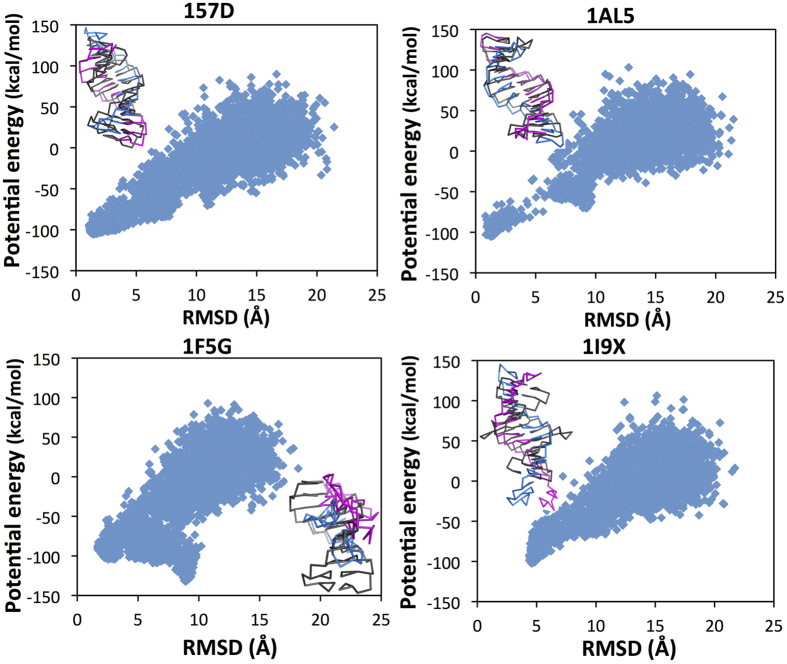
Representative energy landscapes from annealing for two RNAs that are accurately predicted: accurately predicted: 157D and 1AL5 (top), and two RNAs that are unfavorably predicted: 1F5G and 1I9X (bottom). For each RNA, the RACER minimum free energy structure is shown in blue and magenta aligned to the PDB structure shown in black. Five thousand structures over 50 ns are shown for each RNA; each structure is energy minimized before plotting. Note the funnel toward low energy and low RMSD structures. The RMSD of lowest energy structure for 157D is 1.45 Å, 1AL5 is 1.26 Å, 1F5G is 8.91 Å, and 1I9X is 4.56 Å.

The energy vs RMSD landscapes for all 14 RNAs show clear “funnel” shapes skewed toward the native structure. As examples, we present the energy landscapes for two favorably predicted structures (157D and 1AL5, 1.45 Å and 1.26 Å RMSD repectively) in Fig. 4a,b, and the energy landscapes for the two most unfavorably predicted structures (1F5G and 1I9X, 8.91 Å and 4.56 Å RMSD respectively), where the lowest energy structures have large RMSD in Fig. 4c,d.

RACER predicted structures for PDB ID: 157D, 1AL5, 1F5G, and 1I9X are shown in Fig. 4. RACER predicted structures 157D and 1AL5 agree well with experiment (inset in Fig. 4a,b). The RACER predicted structure for 1F5G (8.91 Å RMSD) has collapsed into a torus-like structure, with very little backbone twist (inset in Fig. 4c,d). A possible explanation for this observed behavior is the non-canonical base pairing present in 1F5G. While RACER can capture non-canonical base pairing through the hydrogen bond potential, these hydrogen bonds need further calibration relative to canonical interactions. The RACER predicted structure for 1I9X (4.56 Å RMSD) forms an extended helix compared to the crystal structure. This is likely due to two bases flipped out of the helix in the crystal structure, while RACER incorporates these bases back into the helix. In the crystal structure for 1I9X, several water molecules stabilize these bases. In the RACER model, this stabilization is challenging to capture due to the implicit treatment of solvent via the Debye-Huckel potential.

Additionally, energy landscapes allow us to identify possible meta-stable intermediates, which are high-RMSD (~8 Å) “local” funnels observed in plots for 1DQF and 1QCU. The meta-stable structure of 1DQF at the local minimum, shown in Fig. S2 resembles the toroidal structure observed for 1F5G, but for 1QCU an extended, base stacking meta-stable structure is observed. For 1DQF, the local funnel structure has increased torsional potential energy (~30 kcal/mol) over the global-minimum structure, although both have similar vdW_eff_ and hydrogen bond potentials. For 1QCU, the local funnel structure has a more stabilizing hydrogen bond potential (~−15 kcal/mol) than the global-minimum structure; however, in the global-minimum structure, the Deby-Huckel electrostatics and vdW_eff_ potentials compensate hydrogen bonds to result in an overall more stabilizing intermolecular energy than the local-funnel structure. It is important to note that for both of these RNAs, the RACER global-minimum structure is very close to the experimental structures.

In the process of validating and optimizing our model by energy landscape analysis, we noticed the importance of a dedicated hydrogen bond potential for base paring, as the vdW_eff_ potential is not well suited for distinguishing between base stacking and base pairing interactions^103^. The hydrogen bond potential allows for directional base pairing and helps in separating the base stacking and base pairing interactions effectively.

### Equilibrium Pulling Simulations

#### Experimental free energies

To test RACER, we focused on capturing experimental melting free energies of canonical helices^104^ and hairpins^105^. We used RACER to perform equilibrium pulling simulations, and we compared free energy differences to two sets of experimental thermodynamic data: RNA melting free energies from Turner and coworkers^28^ and folding free energies from single molecule force experiments. Five hairpins of size 10, 10, 12, 14, and 18 nt and five duplexes of size 6, 6, 8, 8, and 10 base pairs were selected from melting free energy experiments, and the TAR RNA hairpin was chosen to compare RACER to single molecule force experiments. Hairpin sequences 30, 11, 33, 47, and 19 from the Supplementary Information of^105^ are referred to here as h1, h2, h3, h4, and h5, and duplex sequences 35, 48, 71, 78, and 90 of^104^ are referred to here as d1, d2, d3, d4, and d5. TAR is a 52 nt, 21 bp hairpin with two internal loops.

In melting free energy experiments, a solution of RNAs of known sequence are heated while measuring UV absorption. As helical and single stranded RNAs absorb light at different wavelengths, the absorption will change over heating as the RNA denatures. By fitting a curve to absorption vs temperature the melting free energy can be determined^106^,^107^,^108^. Turner and co-workers have published a compendium of melting free energies for small RNA motifs and structures using nearest neighbor energy parameters and RNA secondary structure prediction^28^,^104^,^109^. Additionally, we compared our model to RNA single molecule force experiments.

In single molecule force experiments, folded RNA molecules are unfolded by mechanical force using techniques such as optical tweezers or atomic force microscopy. Using the end-to-end extension as a reaction coordinate, the free energy of unfolding can be determined from position vs. time data. A recent single molecule research study of the trans activation response (TAR) element of HIV extracted the free energy of folding at zero force under the assumption of the worm-like chain model^110^. Here we study the same TAR RNA as used in the single molecule force experiments.

Melting and pulling experiments for all RNAs were simulated by umbrella sampling simulations pulling the RNAs apart from their ends (see Fig. S3 for example simulation setup showing end-to-end reaction coordinate). Free energy values were then computed using the Weighted Histogram Analysis Method (WHAM) software distributed by Alan Grossfield^111^. Details of these simulations are included in the Methods section. Although exact energy landscapes at equilibrium for both TAR and melting free energy helices are unknown, folding free energies can be computed according to Eq. 6. The folded free energy, *ΔG*, is found by integrating over all folded conformations at end-to-end extension *r* with free energy *Δω*. Folded free energy is then normalized to volumetric entropy, with standard state volume *V*_*ref*_ of 1660 Å^3^. *kT* is the Boltzmann constant multiplied by temperature (298 K).


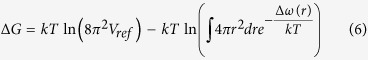


#### Unfolding free energies from RACER MD simulations

**Figure 5:**
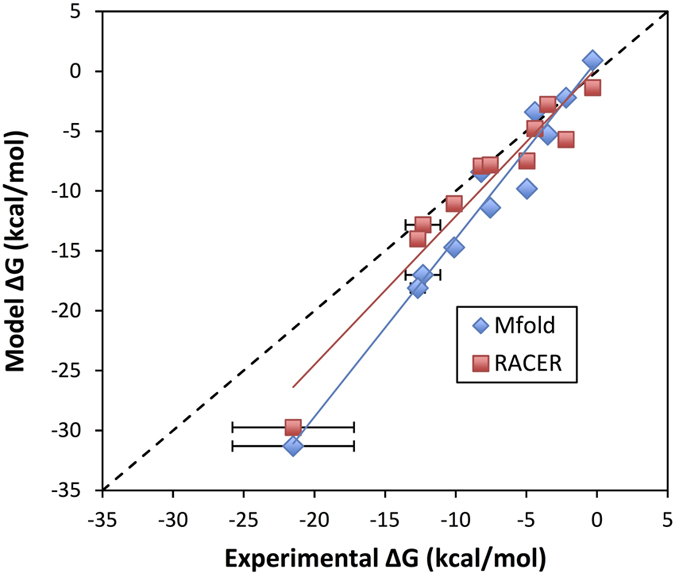
Correlation plot between predicted free energy from RACER and experimental free energy in kcal/mol. RACER simulation predicted free energy is compared with Mfold minimum free energy as well as unity slope (dashed line). RACER and Mfold have the same correlation free energy predictive capability (R^2^ = 0.93 to experimental free energies, experiment), but RACER has a slope closer to unity (slope = 1.2), while Mfold over-stabilizes the free energy of larger RNAs (slope = 1.5). Error bars present on RACER data come from a Monte Carlo bootstrap error analysis as implemented in WHAM by Alan Grossfield^111^ (most errors are within the data point).

The free energies computed from equilibrium pulling MD simulations (WHAM) using RACER are in excellent agreement with experimental measurements, with a correlation coefficient (R^2^) of 0.93 for 11 RNAs tested (Table 2 and Fig. 5). For additional comparison, we also included the melting free energies from Mfold, a widely-used secondary structure prediction program that has been parameterized using the experimental melting thermodynamic data (Mfold predicted structures are shown in Fig. S4). The unfolding free energies evaluated by RACER and Mfold^30^ are presented in Table 2 along experimental values and the length of each MD simulation. The correlation plots for RACER and Mfold show both models have close R^2^ correlation coefficients of 0.93 and 0.96 respectively. However, Mfold’s linear fit has a slightly higher slope (1.5) than RACER (1.2) as Mfold over predicts the stability of the duplexes. Note that RACER is a 3D particle based physical model developed for molecular dynamics simulations, whereas Mfold predicts secondary structures from sequences based on nearest neighbor energy parameters. In RACER we explicitly compute the entropy contributions to the free energy through molecular dynamics sampling.

#### Pulling generated RNA structures

Ensemble model structures for folded states are shown in Figs S5 and S6. In the folded states, TAR, h4 and h5 are observed to form helices while h1–h3 form base pairs and stacking interactions but without regular helical structure. For duplexes, the two RNA strands form canonical base pairs resulting in proper helices. The terminal nucleotides of d5 are observed to break base pairing with one nucleotide rotating out of the helix while the other remains stacked, but this is also observed in experiment^112^.

**Figure 6:**
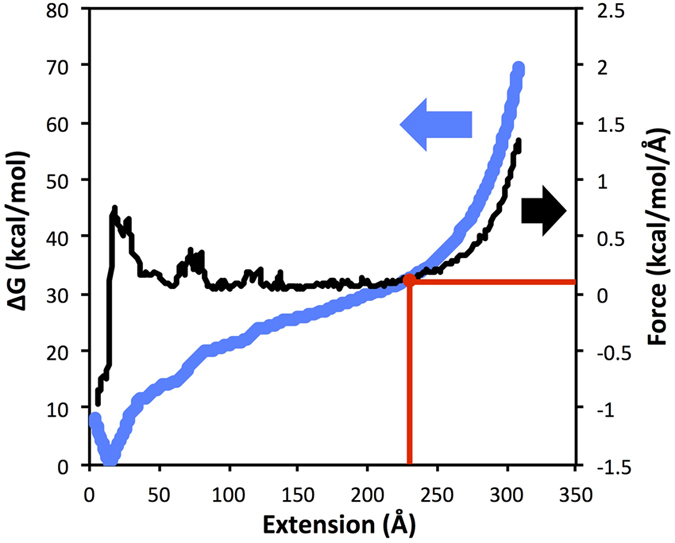
The equilibrium pulling free energy profile (blue) of TAR hairpin computed with WHAM using the RACER model (see Method section details). The unfolded state is determined as the state right before the force (derivative of the free energy, curve shown in black) sharply increases from low (<0.1 kcal/mol/Å) to high due to overstretching. 0.1 kcal/mol/Å and the location of the unfolded state are denoted by the red lines. The calculated folding free energy for TAR is −29.7 ± 0.36 kcal/mol, compared to the experimental value of ≈−21.5 kcal/mol. A 4 Å running average of force (black curve) is shown to eliminate noise.

**Figure 7:**
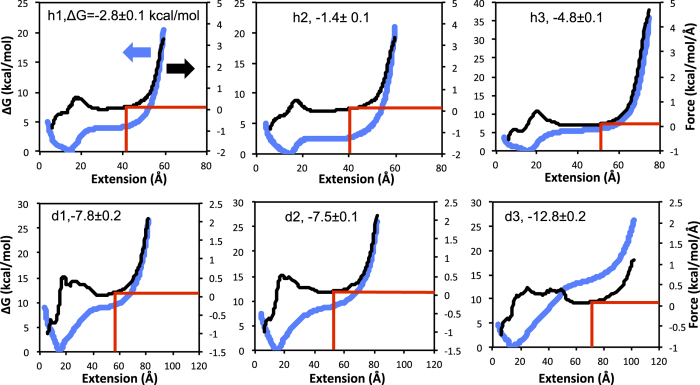
The equilibrium pulling free energy profile (blue) of hairpins h1–h3 (top) and duplexes d1–d3 (bottom) computed with WHAM using the RACER model (h4–h5 and d4–d5 are given in Fig. S7). Umbrella sampling pulling simulations were run for 1 μs for each window, with a 1 Å window separation. The unfolded state is determined as the state right before the force (derivative of the free energy, curves shown in black) sharply increases from low (<0.1 kcal/mol/Å) to high due to overstretching. 0.1 kcal/mol/Å and the location of the unfolded state are denoted by the red lines. The folding free energy (ΔG_f_ kcal/mol) is included for each RNA. A 4 Å running average of force (black curves) is shown to eliminate noise.

In pulling experiments, free energy vs end-to-end extension plots show two distinct energy minima corresponding to folded and unfolded states^113^,^114^,^115^. In the RACER model unfolded (extended) states remain stabilized by vdW_eff_ base stacking interactions, so the location of unfolded free energy is difficult to determine directly from free energy landscapes of RNAs. While the free energy landscapes predicted by RACER show an energy well around the folded state, there is a flat to monotonically increasing curve observed at large extensions (Figs 6 and 7, blue curve, also see Fig. S7). The location of the unfolded state is paramount to computing the folded free energy ΔG using Eq. 6. To determine unfolded state location, we plotted the gradient of the free energy, the ‘force’ as a function of extension (Figs 6 and 7, black curve). From these force vs. extension plots, the predicted free energy of the unfolded state was taken to be the free energy value where the force is very low (~0.1 kcal/mol/Å), i.e. before the RNA reaches the over-stretched regime (Figs 6 and 7, red lines). A 4 Å running average of ‘force’ over extension was used to eliminate noise (Figs 6 and 7). Histogram figures showing equal sampling of the pulling windows are included in Figs S8 and S11. Additionally, the uncertainty of the free energy landscape as computed by a Monte Carlo bootstrap error analysis in the WHAM program by Alan Grossfield^111^ is shown as a range in Figs S12 and S14.

## Discussion

### Statistical potential summary

RACER, a coarse-grained RNA model, can accurately predict native structures and capture RNA folding free energy. The functional forms and parameters in RACER were determined by systematic optimization against native structures and melting free energies for a number of RNA molecules. We found that the statistical potentials^92^ used in the previous model were over stabilizing and the 3D PMFs diverged at long distances. As a result, we treat RNA as a one-dimensional rather than three-dimensional molecule, and use a 1D RDF when fitting to PMFs. Our optimization procedure led us to incorporate a more general effective van der Waals potential energy function (vdW_eff_) to describe the interactions among pseudoatoms.

As a result of implementing a new non-bonded potential energy, we have also reparametrized both electrostatic and hydrogen bond potential energy functions. As the RNA backbone is highly charged, a Debye-Huckel electrostatics term is included for each phosphate pseudoatom; a dielectric of 25 was chosen in order to capture both folded and unfolded RNA structures. A directional hydrogen bond potential was reparametrized in order to accurately distinguish base pairing (hydrogen bond, some vdW_eff_) and base stacking (vdW_eff_) interactions. We found that the hydrogen bond potential was pivotal to accurate folding free energies as both folded and unfolded RNA have base stacking interactions, while only folded RNA have base pairing (hydrogen bond) interactions.

### Thermodynamic summary

For a structure prediction model, thermodynamic accuracy is important to ensure that the energy landscape correctly represents RNAs with varying size and sequence. Our energy landscape analysis suggests that even relatively small RNAs may have complex energy landscapes, and there are many RNA structures at low potential energy. Therefore, explicit consideration of entropy through techniques such as MD is crucial to capture the free energy landscapes of RNA structures.

Folding free energy values for six RNA hairpins of size 10–52 nts and five duplexes of size 6–10 bp were determined by umbrella sampling simulations with WHAM-computed free energy. For hairpins, we determined that umbrella sampling simulations with a reaction coordinate of end-to-end extension is appropriate for capturing folding free energy. For duplexes, the same protocol is found to be appropriate, with the addition of a restraint preventing the single strands from long-lasting intra-strand interactions (e.g. hairpin-like structures). Pulling free energy landscapes of hairpins and duplexes clearly revealed the folded state and we used the gradient (force) of pulling free energy to define the location of the unfolded state.

Given the low computational cost of RACER, over 0.8 ms of umbrella sampling and simulated annealing simulations are presented. Overall, the MD-calculated free energy results using the RNA model are in excellent agreement (R^2^ = 0.93) with experimental folding free energy values while preserving accurate structure prediction. In this work, we present RACER, a novel RNA coarse-grained model that captures both RNA structure and thermodynamics for increased utility to RNA folding investigations.

## Methods

### Mapping from all-atom to coarse-grained structures

A notable feature of our model is the ability to map to and from all-atom experimental crystal structures. Each of our model’s pseudoatoms represents an atomic site in nucleotides; for example, the sugar pseudoatom is assigned the C4’ atom position on ribose. Moreover, our model captures the planarity of the nucleobase with three pseudoatoms. Given a novel (structure undetermined) RNA sequence, our model can first predict the three-dimensional structure in coarse-grained coordinates and then map to all-atom coordinates with further minimization, producing an equivalent to an all-atom experimentally determined structure. As a result, our RNA model is well suited to perform multiscale simulations in the future.

### Pulling methods

Melting and pulling experiments are modeled by using umbrella simulations pulling the RNA molecule apart from its terminal ends. A harmonic potential of 1 kcal/mol/Å^2^ spring constant is used to restrain the RNA ends at the sugar pseudoatoms (C4’ sugar atomic site). Simulation extensions ran from 5.5 Å up to fully extended lengths (59.5, 76.5, 86.5, 106.5, and 307.5 Å for 10, 12, 14, 18, and 52 nt hairpins assuming 5.9 Å per nt contour length) with a spacing of 1 Å between windows.

Duplexes are similarly pulled apart from the sugar pseudoatoms at one terminal end with a 1 kcal/mol/Å^2^ spring constant; the other terminal end is restrained between two terminal sugar pseudoatoms with a 1 kcal/mol/Å^2^ spring constant. Duplex extensions ranged from 5.5 Angstroms up to fully extended lengths (80.5, 100.5, and 124.5 Angstroms for 6, 8, and 10 base pair duplexes respectively) with umbrella window spacing of 1 Å. For the duplexes and shorter hairpins of size 10 and 18 nt, 1 μs of Molecular Dynamics was run for each window. For the TAR hairpin, 100 ns was found to be sufficient given the longer end-to-end extension (more windows) needed. We used a 4 fs time step for pulling simulations. From the umbrella simulations, the free energy landscapes were computed by the Weighted Histogram Analysis Method^116^ (WHAM) using the program distributed by Alan Grossfield^111^.

### Computational efficiency of the RACER Model

All annealing and pulling simulations (total of 0.86 ms) were computed on a local computer cluster. For all simulations discussed below a 4 fs time step was used, and the CPUs used are an early generation Intel Xeon E5345 2.33 GHz CPU. Using one CPU core for each simulation, 1 μs of simulation of the 10 nt hairpin h1 took 22 hours, 1 μs of simulation of the 18 nt hairpin h3 took ~60 hours, and 100 ns of simulation of the 52 nt hairpin TAR took ~48 hours. Additionally, 1 μs simulation of duplex d35 required 30 hours, while 1 μs for duplex d90 required 74 hours. Recently, RACER has been implemented with OpenMP allowing parallelization to multiple cores. In the future, we will implement our model on GPUs, using the software package OpenMM^117^. Implementation of RACER on GPUs will allow for even better efficiency. As a result of the improved computational efficiency offered by the coarse-graining, it will be possible to simulate RNAs at physiologically relevant timescales.

### Implementation and parameters

The TINKERMD implemented RACER model is available free of charge at http://biomol.bme.utexas.edu/tinker-openmm/index.php/TINKER-OPENMM:Development-rna. The parameters and conversion programs are included in the distribution. Conversion tutorials are posted online at http://biomol.bme.utexas.edu/tinker-openmm/index.php/TINKER-OPENMM:Tutorials-rna.

## Additional Information

**How to cite this article:** Bell, D. R. *et al*. Capturing RNA Folding Free Energy with Coarse-Grained Molecular Dynamics Simulations. *Sci. Rep.* **7**, 45812; doi: 10.1038/srep45812 (2017).

**Publisher's note:** Springer Nature remains neutral with regard to jurisdictional claims in published maps and institutional affiliations.

## Supplementary Material

Supplementary Information

## Figures and Tables

**Table 1:** Predicted (minimum energy) RMSD values compared to Protein Data Bank (PDB) structures from simulated annealing.

PDB ID	157D	1AL5	1DQF	1F5G	1I9X	1KD5	1LNT	1QCU	1ZIH	2AO5	2JXQ	2K7E	353D	472D	Avg.
RMSD (Å)	1.45	1.26	3.04	8.91	4.56	2.88	2.65	0.96	3.11	1.83	1.13	3.58	2.24	3.47	2.93 ± 0.54

**Table 2:** Unfolding free energy values for RNAs from experiment (Expt.), Mfold predicted, and RACER predicted.

Hairpin	Length (nt)	Expt. ΔG (kcal/mol)	Mfold ΔG (kcal/mol)	RACER ΔG	Length per window (kcal/mol)
h1	10	−3.5 ± 0.3^105^,^118^	−5.3	−2.8 ± 0.12	1 μs
h2	10	−0.3 ± 0.1^105^,^119^	+0.9	−1.4 ± 0.12	1 μs
h3	12	−4.4 ± 0.2^105^,^120^	−3.4	−4.8 ± 0.14	1 μs
h4	14	−2.2 ± 0.08^105^,^121^	−2.2	−5.7 ± 0.15	1 μs
h5	18	−8.2 ± 0.2^105^,^122^	−8.4	−7.9 ± 0.22	1 μs
TAR	52	≈−21.5 ± 4.3^110^	−31.3	−29.7 ± 0.36	0.1 μs
**Duplex**	**Length (bp)**				
d1	6	−7.56 ± 0.3^104^	−11.4	−7.8 ± 0.15	1 μs
d2	6	−4.95 ± 0.2^104^	−9.8	−7.5 ± 0.14	1 μs
d3	8	−12.32 ± 1.2^104^	−17.0	−12.8 ± 0.17	1 μs
d4	8	−10.11 ± 0.2^104^,^106^	−14.7	−11.1 ± 0.18	1 μs
d5	10	−12.69 ± 0.5^104^	−18.1	−14.0 ± 0.19	1 μs
					Total: 860 μs

Molecule length in nucleotides or basepairs and the simulation length per window are also shown. Error is take from a Monte Carlo bootstrap error analysis as implemented in the WHAM program by Grossfield^111^.
