# Supplementary material for: Capturing RNA Folding Free Energy with Coarse-Grained Molecular Dynamics Simulations
Source: Sci Rep. 2017 Apr 10;7:45812. doi: 10.1038/srep45812 (PMC5385882; doi:10.1038/srep45812)
Supplement: Supplementary Information [file srep45812-s1.pdf]

Supplementary Information

"Capturing RNA Folding Free Energy with Coarse-Grained Molecular Dynamics Simulations"

David R. Bell[1], Sara Y. Cheng[2], Heber Salazar[1], and Pengyu Ren[1*]

[1] Department of Biomedical Engineering, University of Texas at Austin, Austin, Texas 78712, United States
[2] Department of Physics, University of Texas at Austin, Austin, Texas 78712, United States

* Corresponding author: pren@mail.utexas.edu

Table of Contents Pg

1. Fig. S1 Hydrogen bond potential 1
2. Table S1. Annealing energy landscapes 2-4
3. Fig. S2 Annealing funnel feature structures 5
4. Fig. S3 Pulling simulation system configuration 6
5. Fig. S4 Mfold predicted structures for pulling simulations 7
6. Fig. S5 Equilibrium extension structures for hairpins 8
7. Fig. S6 Equilibrium extension structures for duplexes 9
8. Fig. S7 Pulling free energy landscape for helics h4-h5 and duplexes d4-d5 10
9. Fig. S8-S11 Umbrella sampling histograms 11-14
10. Fig. S12-S14 Pulling free energy landcsapes with error 15-17
11. Debye-Huckel parameterization 18
12. Fig. S15 Debye-Huckel parameters effect on RMSD and potential 19
13. Torsion parameterization 20-21
14. Fig. S16 Example correlation between torsion angles 21
15. Hydrogen bond details 22
16. Table S2 Base-pairing vs. Stacking 22
17. Directionality and derivative of hydrogen bond potential 23
18. Fig. S17 Derivative of hydrogen bond potential 23-25
19. References 25

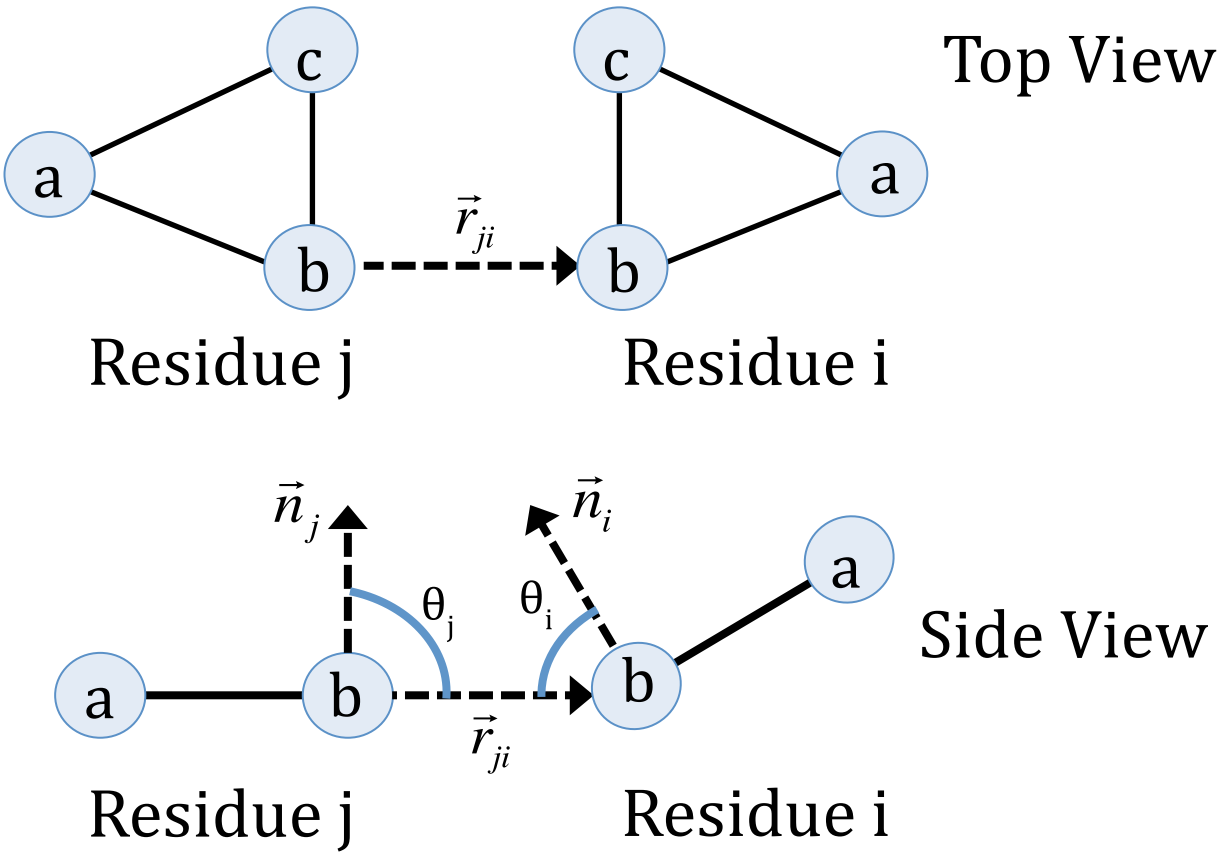

$$E_{hb} = -\frac{\varepsilon_{hb,max}}{2}(1-\cos(\alpha_k))\left(\frac{\sigma_{hb,eq}}{|\overrightarrow{r_{ji}}|}\right)^3$$

$$\alpha_k = 2(\theta_i+\theta_j)-\pi, \qquad \frac{\pi}{2} < (\theta_i+\theta_j) < \frac{3\pi}{2}$$

$$\theta_j = \cos^{-1}\left(\frac{\overrightarrow{n_j}\cdot\overrightarrow{r_{ji}}}{|\overrightarrow{n_j}||\overrightarrow{r_{ji}}|}\right), \qquad \overrightarrow{n_j} = \overrightarrow{r_{jab}}\times\overrightarrow{r_{jcb}}$$

Fig. S1. Hydrogen bond potential diagram and equations. $\overrightarrow{n_i}$ and $\overrightarrow{n_j}$ are the vectors normal to the plane of residues i and j respectively. $\overrightarrow{r_{jab}}$ is the vector from atom b to atom a on residue j and $\overrightarrow{r_{jcb}}$ is the vector from atom c to atom a on residue j. $\overrightarrow{r_{ji}}$ is the vector between hydrogen bonding atoms of residues j and i. θ_i and θ_j are the angles between the respective normal vectors and vector $\overrightarrow{r_{ji}}$.

Table S1. Simulated annealing energy landscapes for 14 PDB structures. PDB ID is stated at the top of each plot. The total potential energy as a function of RMSD to the PDB structure are shown. For annealing protocol, see main text.

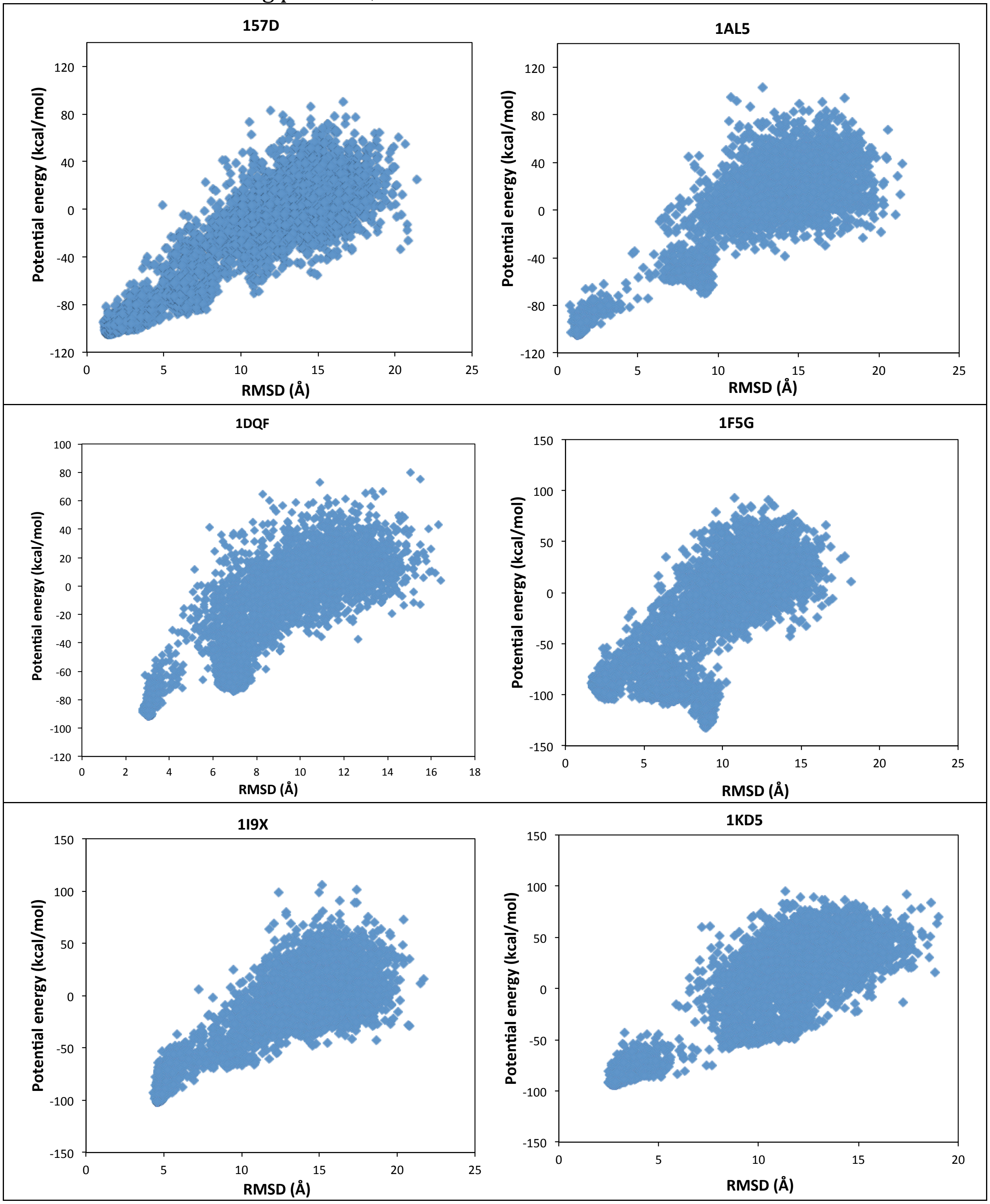

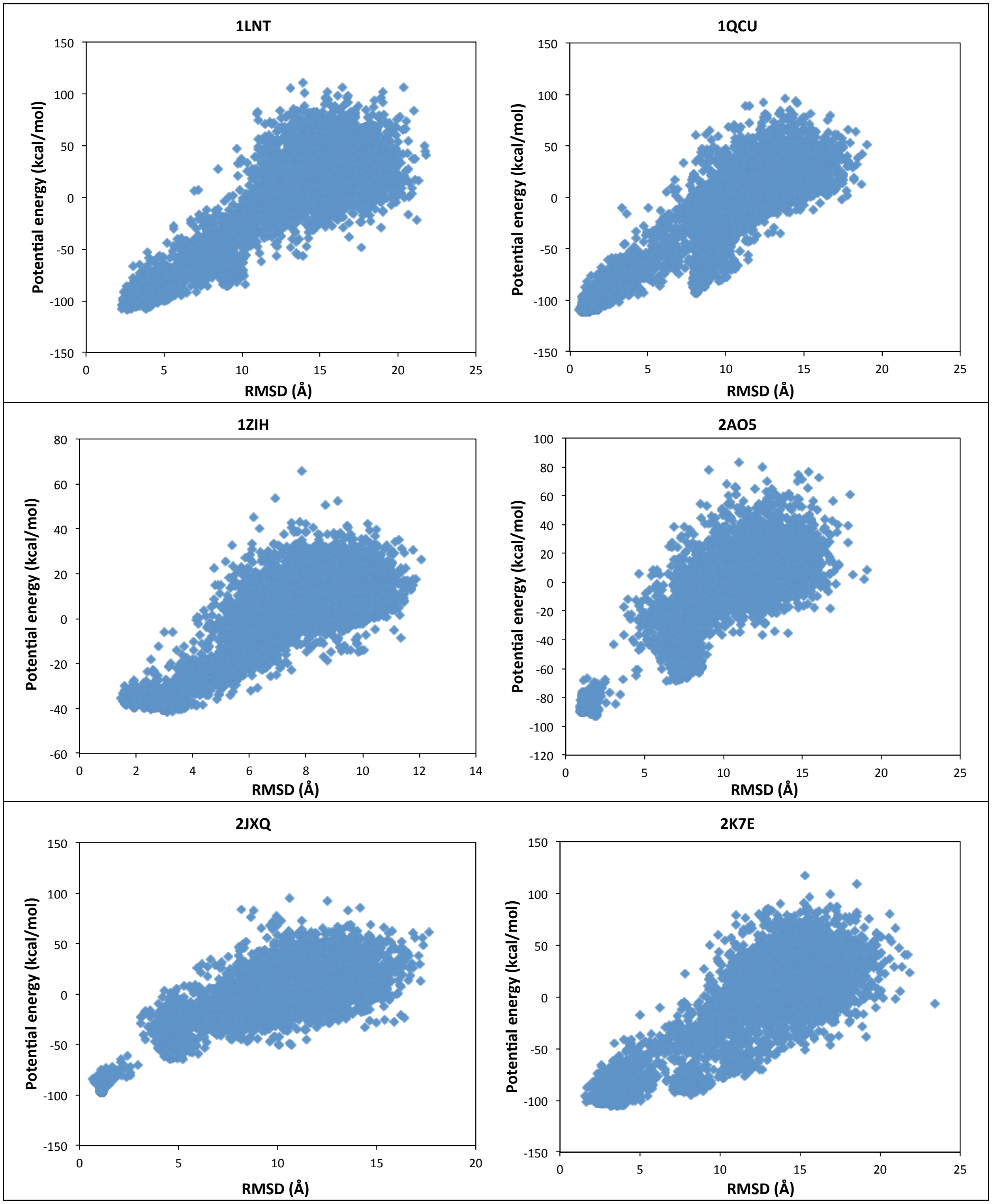
1LNT
1QCU
1ZIH
2AO5
2JXQ
2K7E
Potential energy (kcal/mol)
RMSD (Å)
150
100
50
0
-50
-100
-150
0
5
10
15
20
25
80
60
40
20
-20
-40
-60
2
4
6
8
12
14
-80
-120

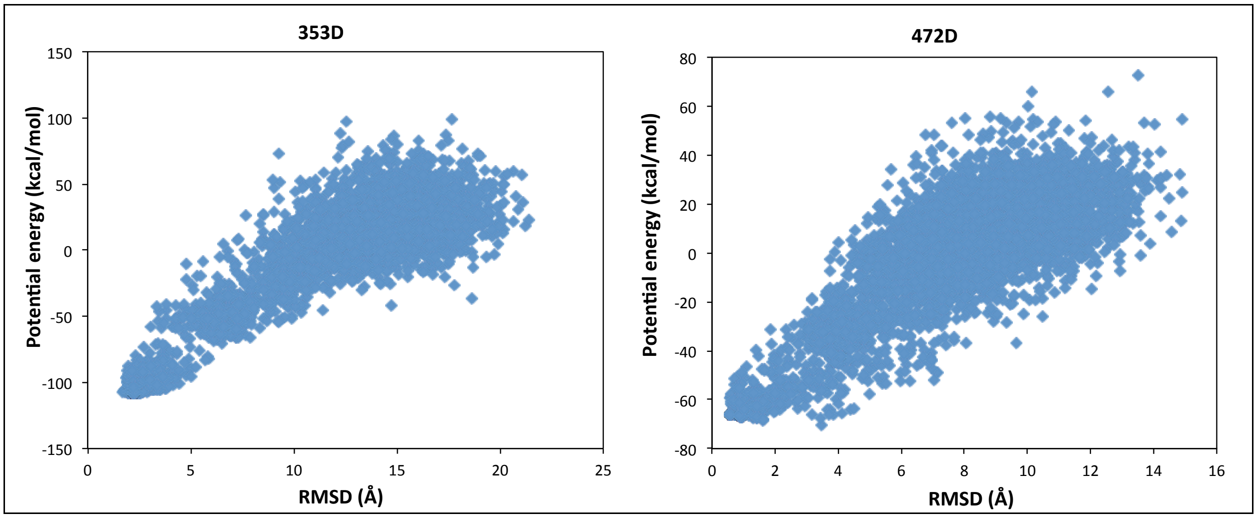

353D
Potential energy (kcal/mol)
150
100
50
0
-50
-100
-150
0
5
10
15
20
25
RMSD (Å)
472D
Potential energy (kcal/mol)
80
60
40
20
0
-20
-40
-60
-80
0
2
4
6
8
10
12
14
16
RMSD (Å)

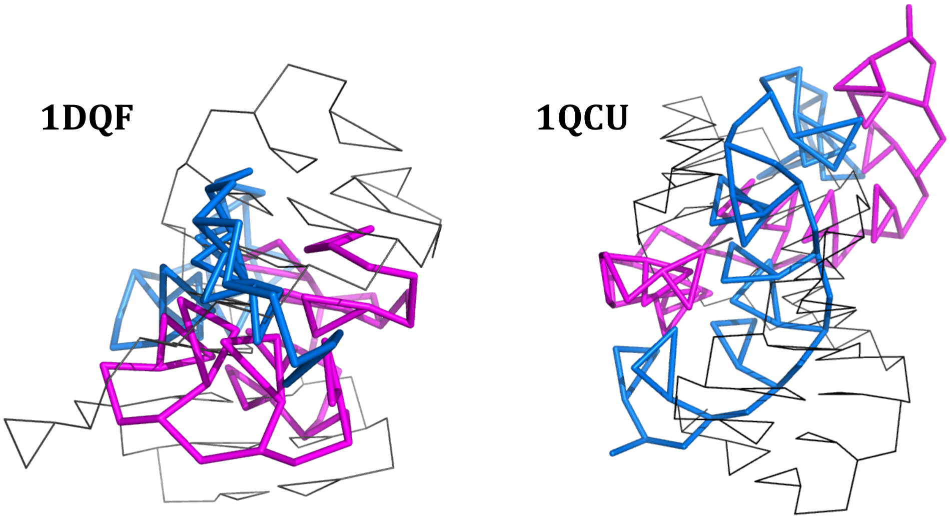

Fig. S2. Annealing structures taken from bottom of funnel feature: 1DQF (left) and 1QCU (right). Annealing structures are colored blue and magenta while experimental structures are colored black. Note the extended, base-stacking structure of 1QCU.

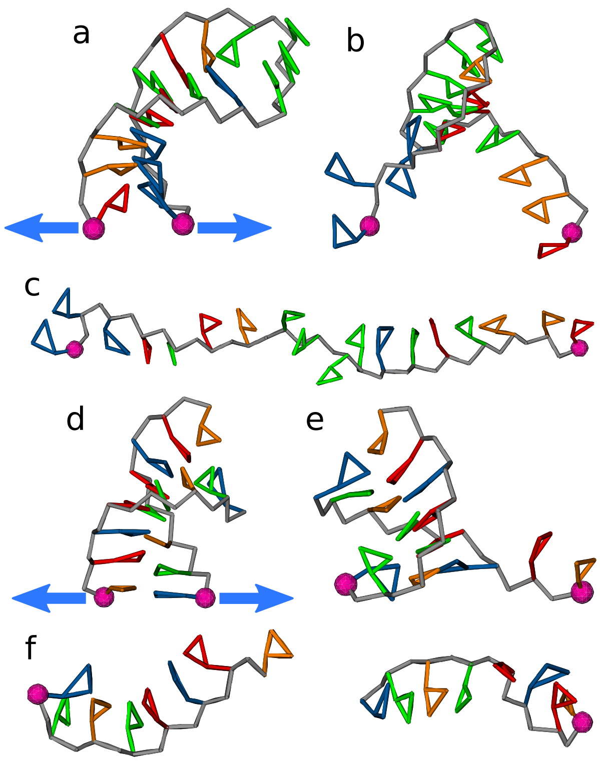

Fig. S3. Pulling simulation setup of Hairpin h3 (a-c) and duplex d78 (d-f). The RNAs were pulled along the reaction coordinate of end-to-end extension (marked by large magenta spheres) using umbrella simulations. The magenta spheres at the strand ends represent the sugar pseudoatoms that were restrained in umbrella simulations. (a, d) native end-to-end extension (b, e) partially denaturing extensions and (c, f) and unfolded/melted extensions. Note that in the folded structures, base stacking and base pairing interactions exist, while in unfolded or melted structures, only base stacking interaction exists. Gray bonds are backbone atoms while red, orange, green, and blue bonds are A, C, U, and G nucleobases respectively.

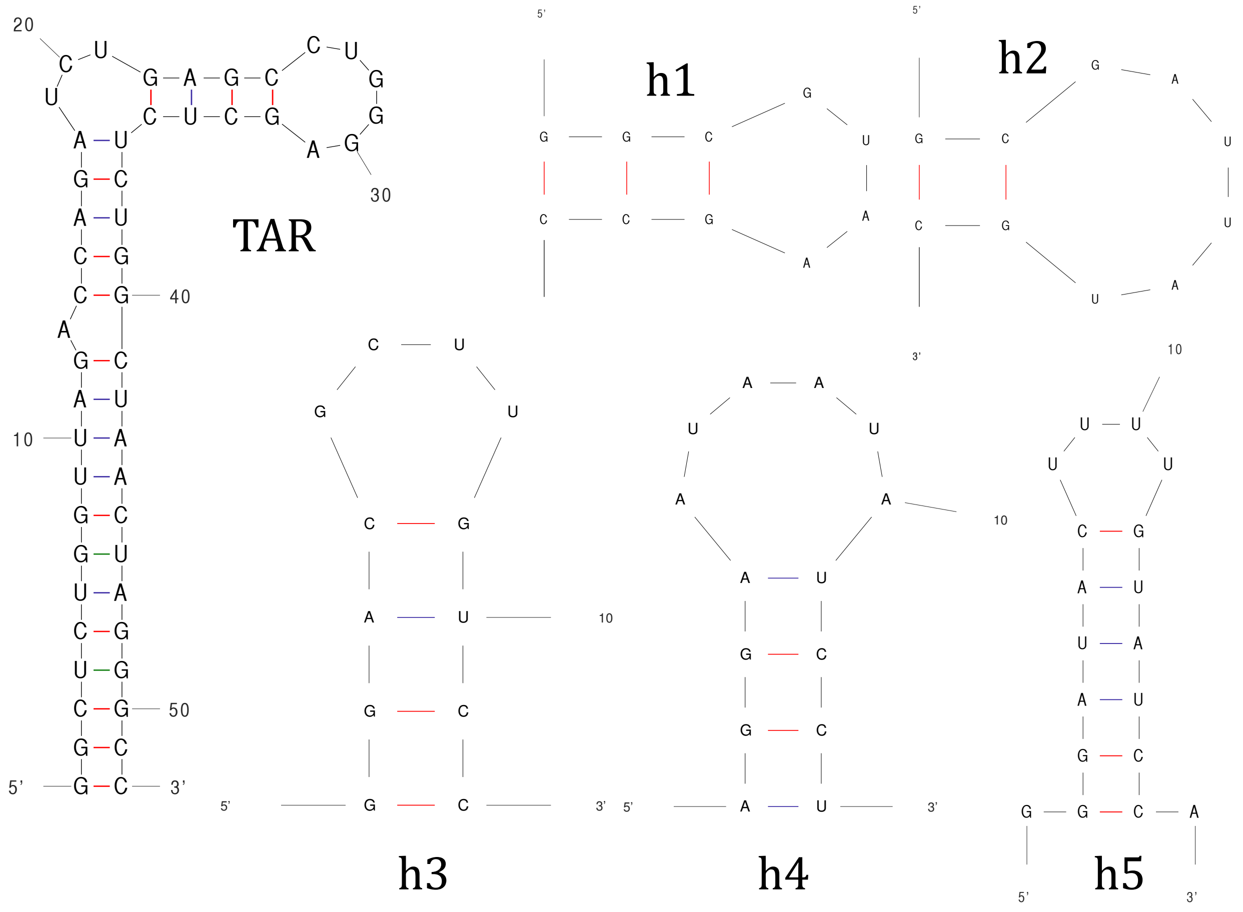

Fig. S4. Mfold predicted minimum free energy secondary structures for the hairpins reported: TAR (left), and Turner hairpin sequences h1 (top, middle), h2 (top, right), h3 (bottom left), h4 (bottom, middle), and h5 (bottom, right).

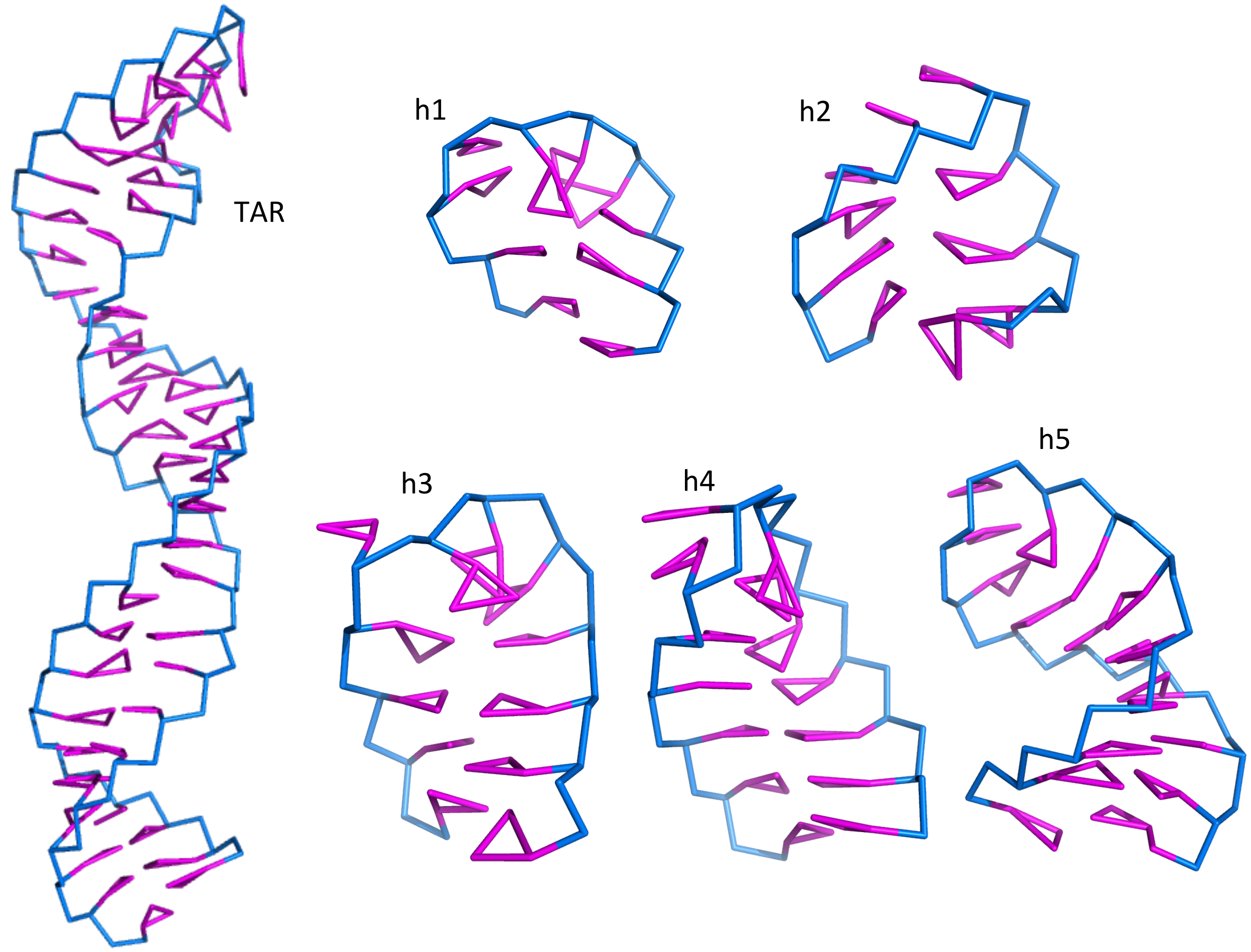

Fig. S5. Model structures of hairpins used for pulling sequences. Structures are taken from the ensemble of equilibrium end-end extension structures.

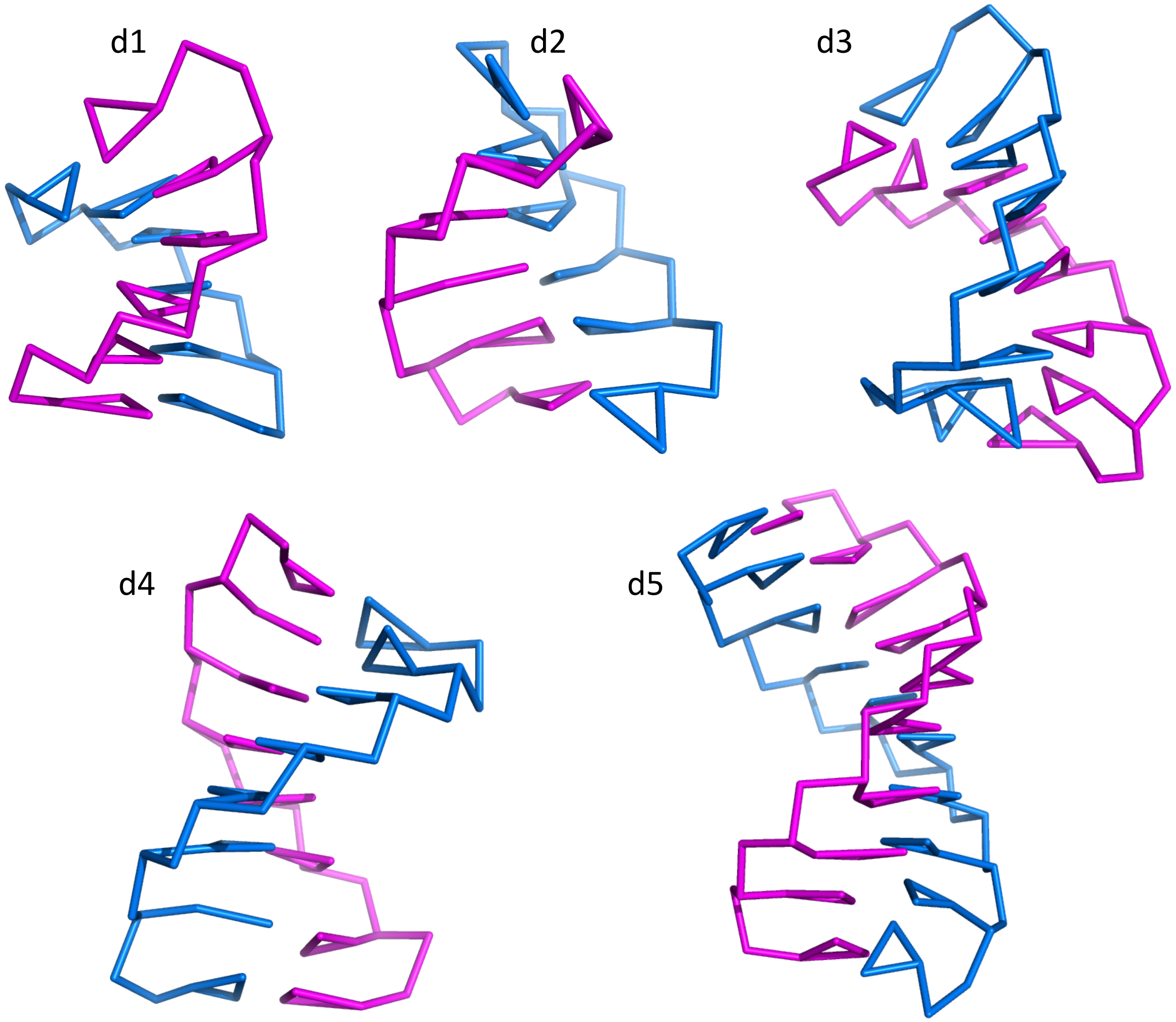

Fig. S6. Model structures of duplexes used for pulling simulations. Structures are taken from the ensemble of equilibrium structures.

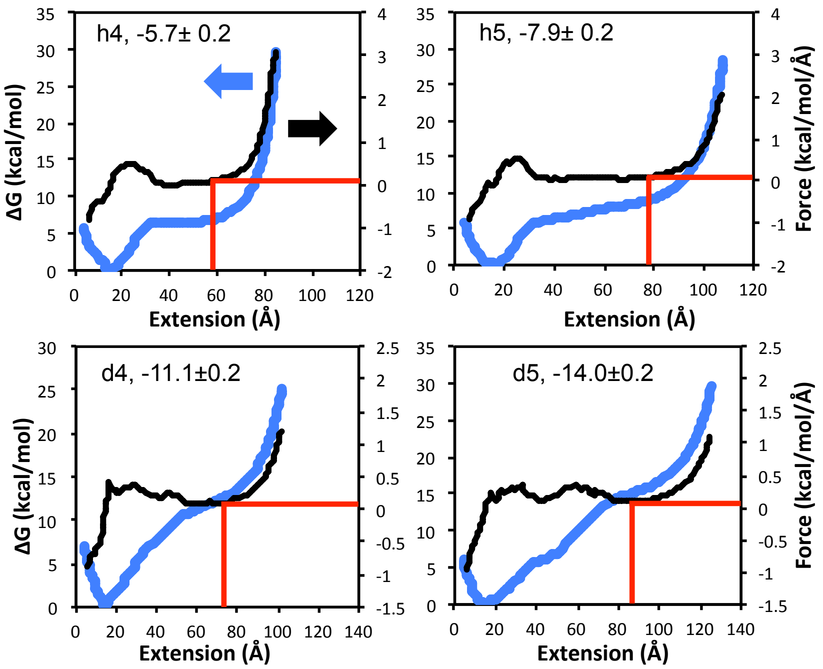

Fig. S7. The equilibrium pulling free energy profile (blue) of hairpins h4-h5 (top) and duplexes d4-d5 (bottom) computed with WHAM using the RACER model. Umbrella sampling pulling simulations were run for 1μs for each window, with a 1Å window separation. The unfolded state is determined as the state right before the force (derivative of the free energy, curves shown in black) sharply increases from low (< 0.1 kcal/mol/Å) to high due to overstretching. 0.1 kcal/mol/Å and the location of the unfolded state are denoted by the red lines. The folding free energy (ΔG_f kcal/mol) is included for each RNA. A 4Å running average of force (black curves) is shown to eliminate noise.

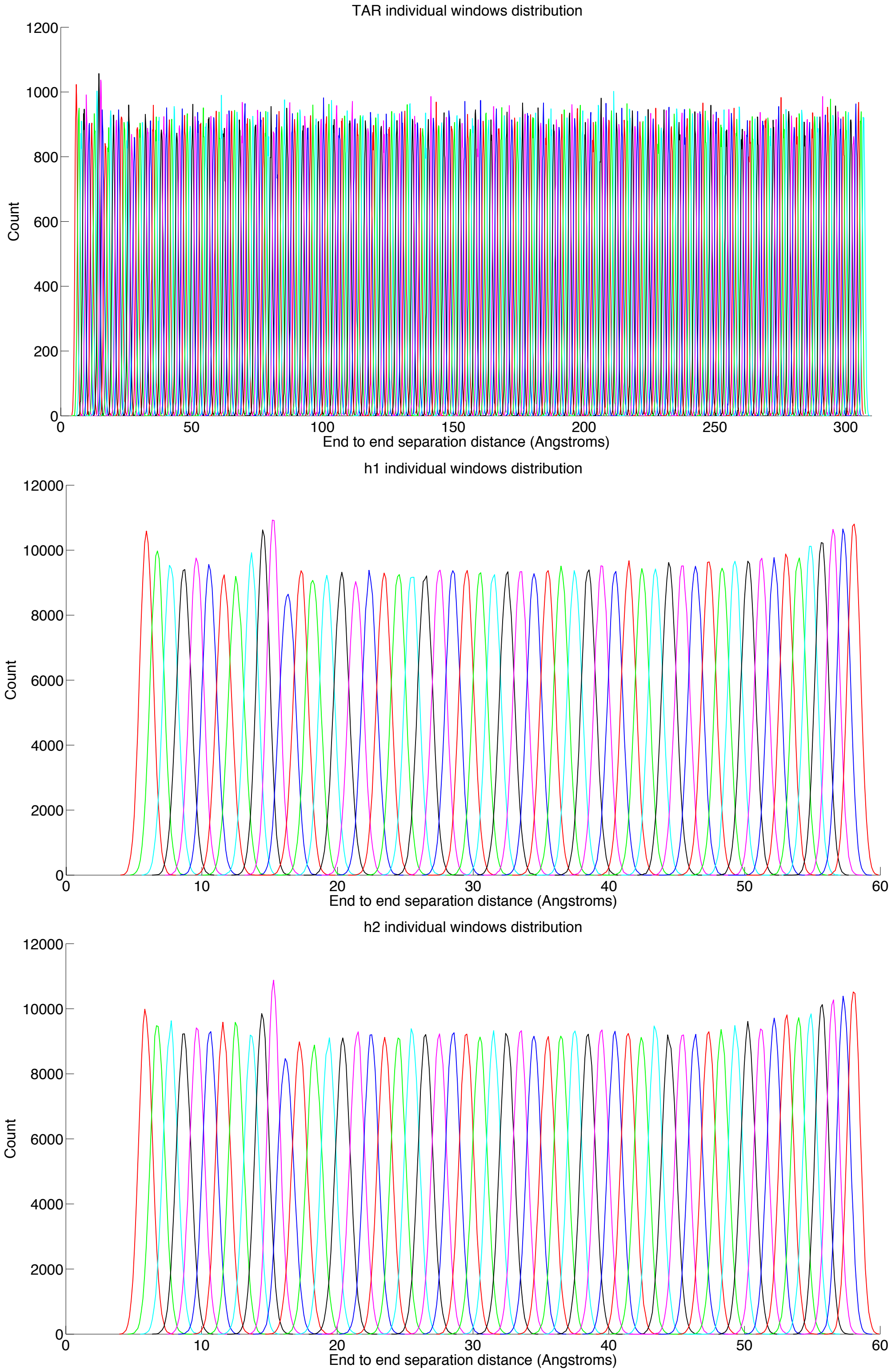

Fig. S8. Sampling distribution of each umbrella sampling window for hairpins TAR (top) and h1-h2. The separation distance between windows was 1Å for all RNAs.

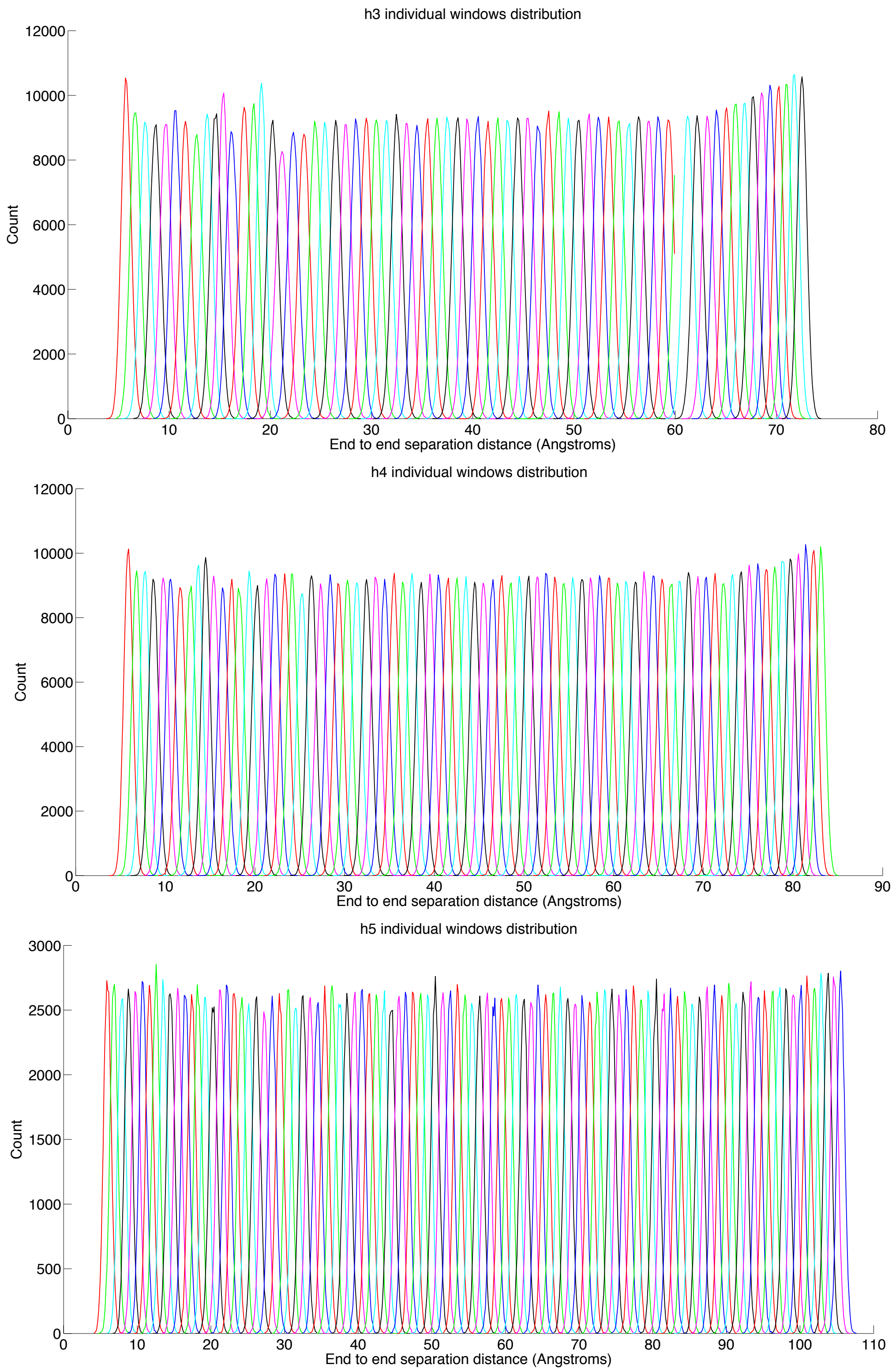

Fig. S9. Sampling distribution of each umbrella sampling window for hairpins h3-h5. The separation between windows is 1Å.

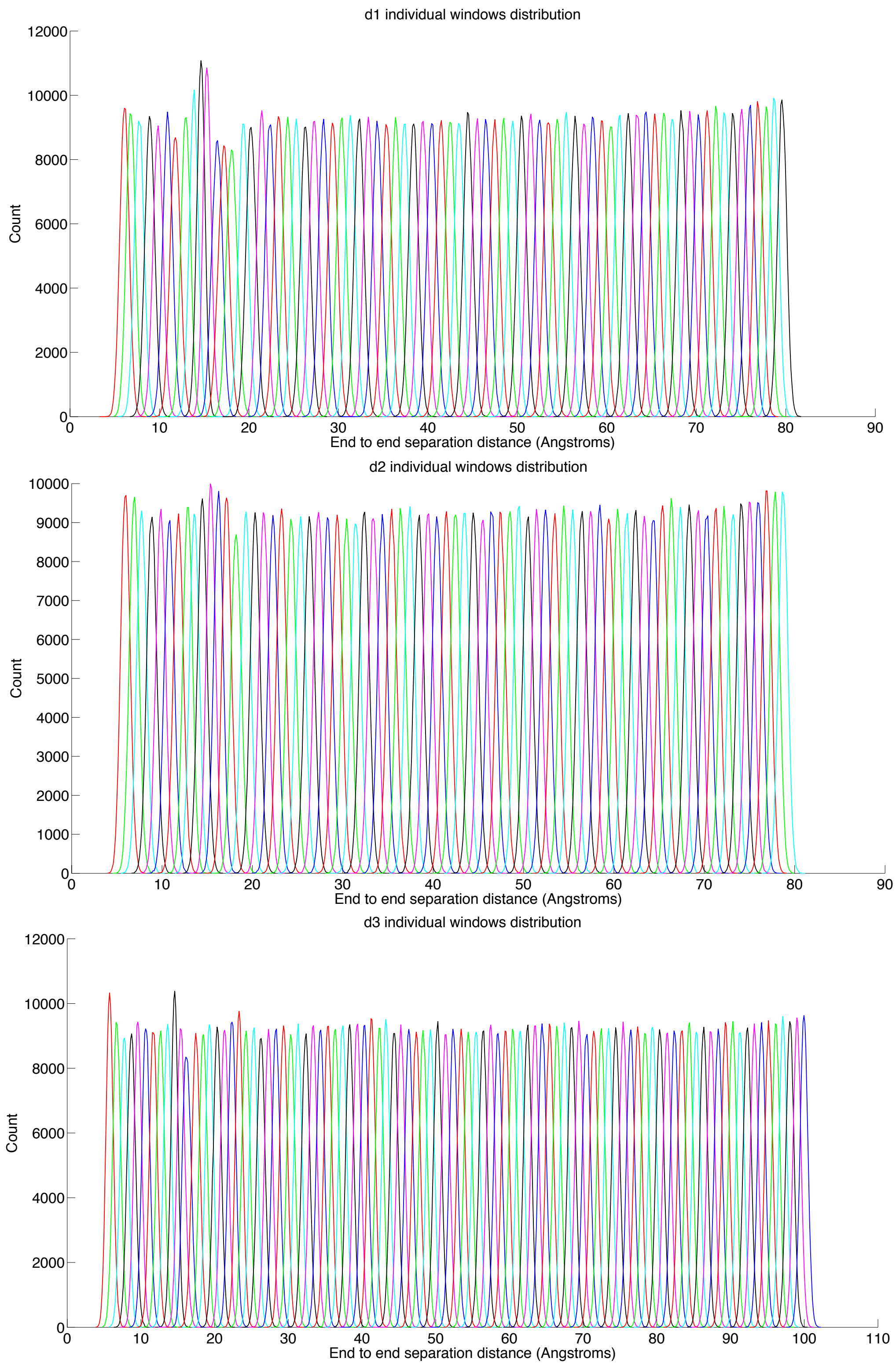

Fig. S10. Sampling distribution of each umbrella sampling window for duplexes d1-d3. The separation between windows is 1Å.

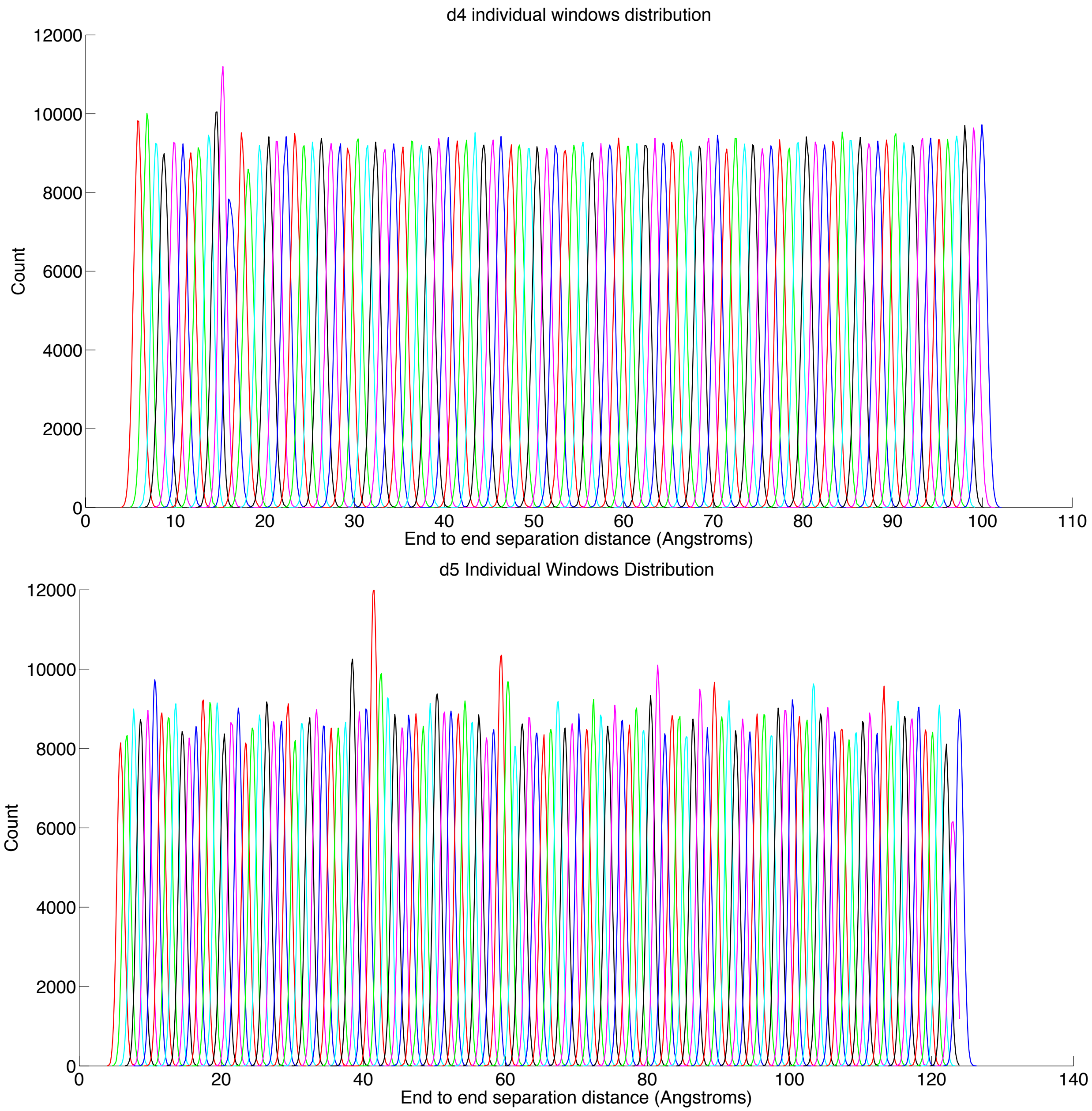

Fig. S11. Sampling distribution of each umbrella sampling window for duplexes d4-d5. The separation between windows is 1Å.

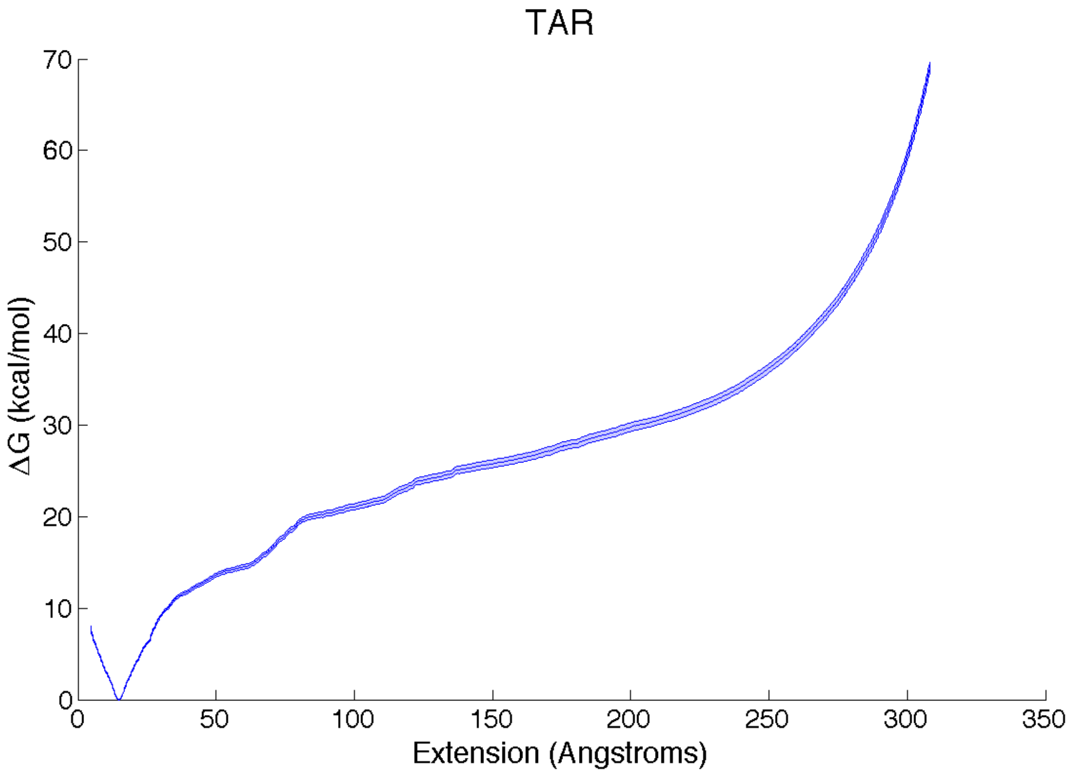

Fig. S12. TAR pulling free energy landscape with computed error shown as range. Error is takem from a Monte Carlo bootstrap error analysis as implemented in the WHAM program by Grossfield[1]. The RACER predicted free energy is -19.7±1.39 kcal/mol; the experimental value is ≈-21.5 kcal/mol.

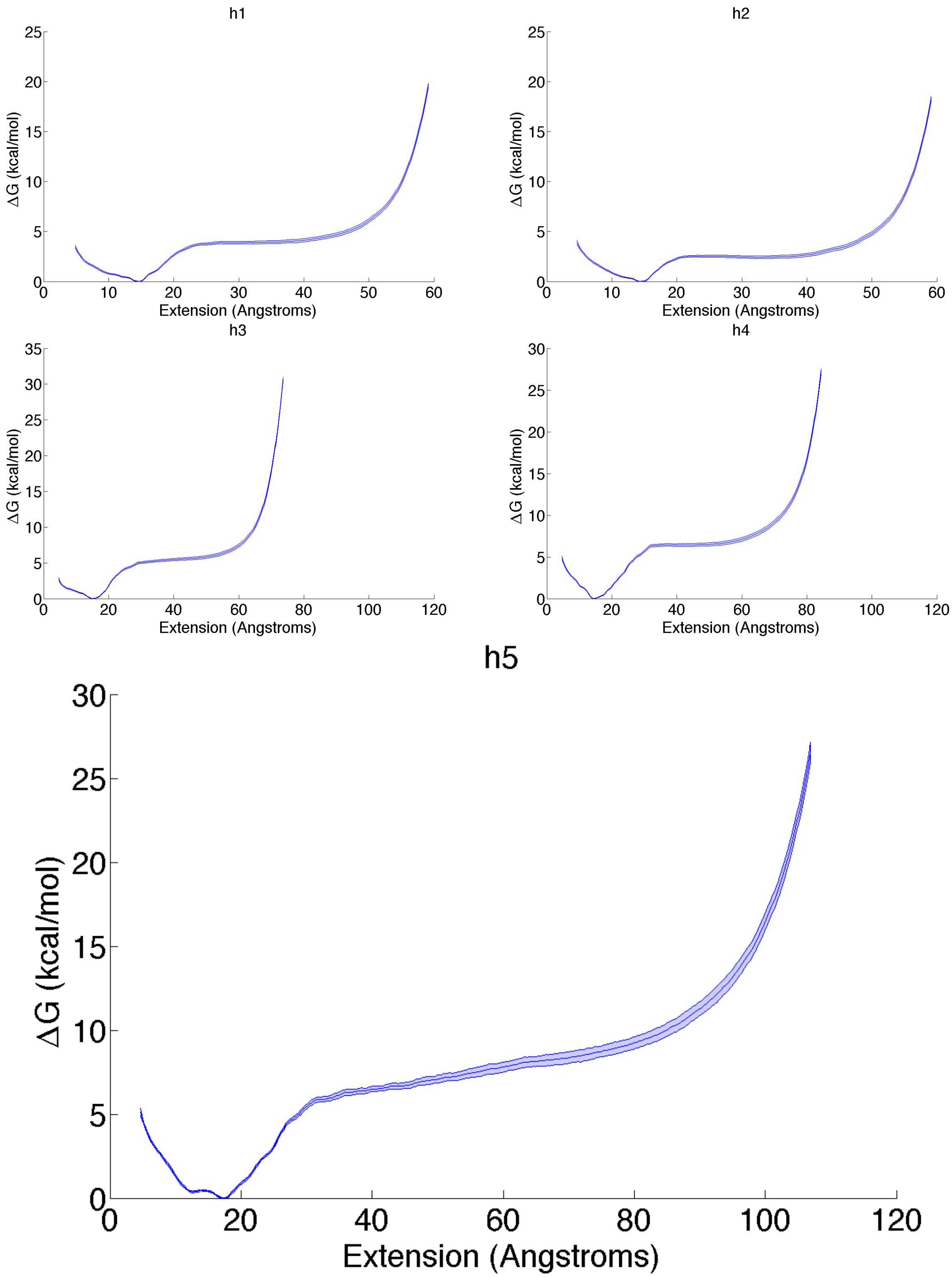

Fig. S13. Hairpins h1-h5 pulling free energy landscapes with computed error shown as range. Error is taken from a Monte Carlo bootstrap error analysis as implemented in the WHAM program by Grossfield[1].

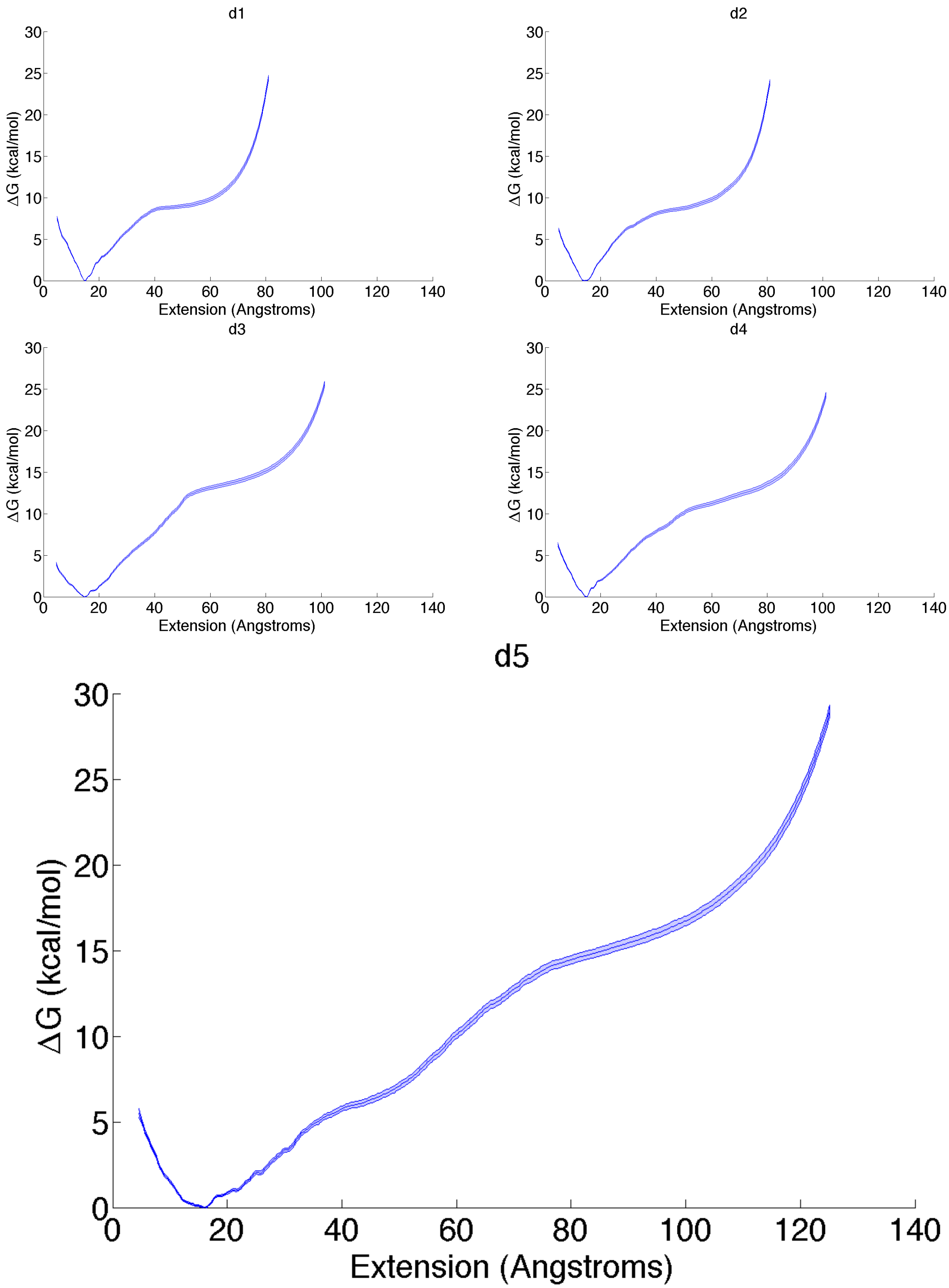

Fig. S14. Duplexes d1-d5 pulling free energy landscapes with computed error shown as range. Error is taken from a Monte Carlo bootstrap error analysis as implemented in the WHAM program by Grossfield[1].

Debye-Huckel parameterization. The electrostatics potential also contributes to non-bonded interactions and should be included in fitting to the PMF curves. We used the same Debye-Huckel electrostatics term shown in Eq. 4 for our model. In RNA nucleotides, only the phosphate group is net-charged and correspondingly, only the phosphate pseudoatom in our model has a charge of -1.0*e*. The Debye-Huckel potential is purely repulsive, so this must be balanced by attractive terms with the vdW_{eff} potential, when fitting to the phosphate nonbonded PMF. Note that this potential incorporates an implicit solvent effect in addition to Coulomb interactions. During optimization, the Debye-Huckel potential was fit concomitantly to the vdW_{eff} potential for both structure and energy. In order to select an optimal dielectric constant and Debye-length for our model, we analyzed the effects of varying the dielectric constant and Debye-length on the RMSD and Pearson R^2 correlation of our model RMSD and experimental energies for 14 PDB structures listed in Table 1. As seen in Fig. S14, our selection of a dielectric constant of 25 and a Debye length of 10 Angstroms corresponds to both low RMSD and high R^2 correlation. Typical dielectric constants range from ~78 for pure water[2] to <5 for folded proteins[3,4]. Small RNAs are much more exposed than compact proteins. Though the dielectric constant will vary over RNA structure, a dielectric value of 25 is a reasonable compromise between folded and unfolded states. Whereas the dielectric is a proportionality constant, the Debye-length ξ acts as a decay constant, describing how quickly the electrostatic energy decreases over distance. If we assume a monovalent salt concentration of 0.1 M, the Debye-length is approximately 9.6 Angstroms[5], so the Debye-length of 10 Angstroms used here is appropriate.

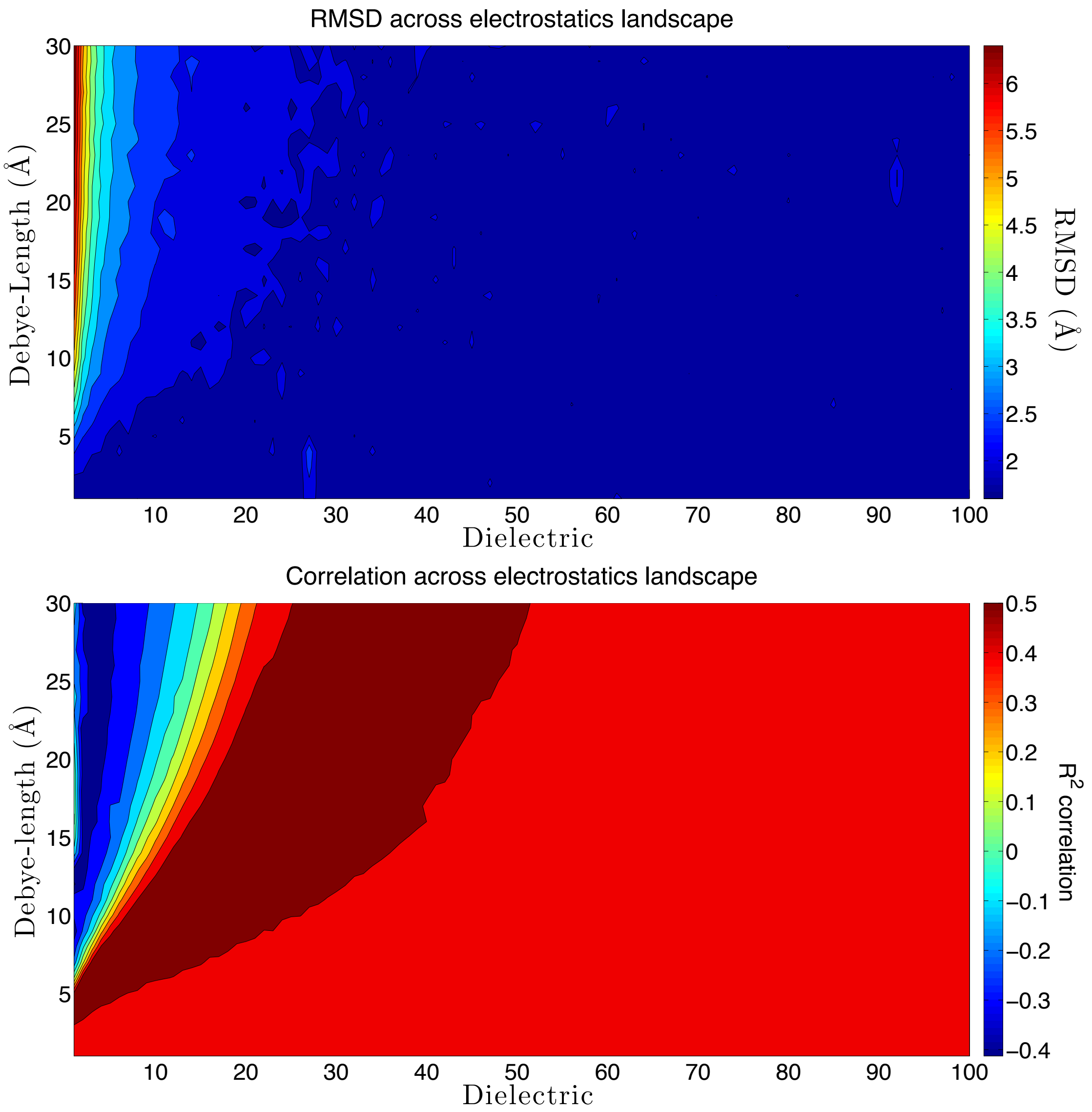

Fig. S15. RMSD (top) and Pearson R^2 correlation coefficient (bottom) values as a function of Dielectric and Debye-Length (Å). The RMSD value is the average of 14 PDB structures averaged over 5ps molecular dynamics simulation. Pearson product moment correlation coefficient (R^2) is between RACER model potential energy after minimization and experimental melting free energies for a set of 90 RNA sequences taken from ref[6].

Torsion parameterization. Parameterization of model torsion interaction required consideration of double counting (overlap between the nonbonded and torsional terms) and formation of non A-form helices. Similar to nonbonded interactions, when torsion parameters were naively fit to the PDB structure-statistics derived PMF, the torsion potential was excessively large (3x bending and angle potential energies). To better understand this, we studied the residue-level torsion interactions and determined that for the raw-PMF torsion parameters, torsion interactions are at least double and in some cases quadruple counted since multiple torsional terms are describing the exactly same rotation. For instance, in the backbone S-P' pseudoatom connection, there exists both torsion terms PSP'S' as well as C*SP'S', where prime(') represents the adjacent nucleotide and C* represents the pseudoatom type CU or CG. Although these torsion terms depict distinct chemical structures, they are not independent. Further, the S-C* pseudoatom connection is quadruple counted, with interactions PSC*B1, PSC*B2, B1C*SP', and B2C*SP' where B1 and B2 represent two separate base atoms. A correlation figure of 2 S-C* torsions is shown in Figure S15. Note that if the S-C* torsions were not correlated, Figure S15 would be a uniform blue rectangle; the concise sampled area is a clear indication of correlation. To address the above issue, we reduced the k_n torsional force constants to half (or quarter in some cases) of their PMF-fit values. Another important consideration is that the Boltzmann inversion from structural-statistics assumes torsion or pair-wise PMF is independent of all other interactions. What the "torsion" PMF captures is a combination of atomic repulsion-dispersion and electrostatic interactions. In our model vdW_{eff} interactions between 1-4 bonded pseudoatoms are not computed. However, we still found considerable interplay between torsion and vdW_{eff} interactions, and the balance of these terms must be carefully

accounted for in the parameterization of effective nonbonded potential. Ultimately, by consideration of double counting and structural flexibility, we optimized the torsion potential to adequately depict intra-strand rotation.

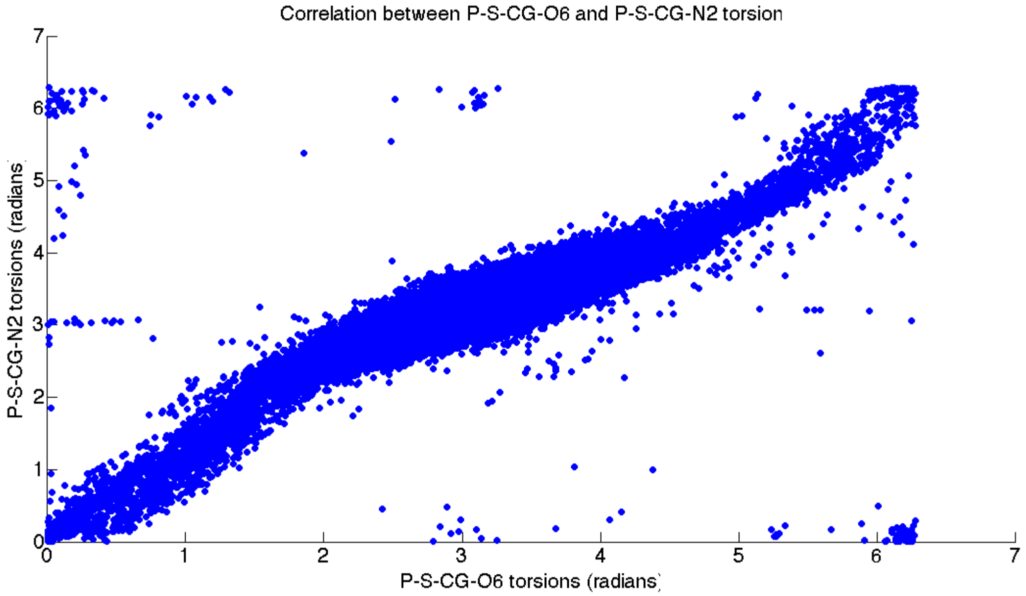

Fig. S16. Correlation of torsions P-S-CG-O6 and P-S-CG-N2 from Guanosine nucleotides. Over 70,000 PDB Guanosine nucleotides were sampled; uncorrelated torsions would yield a completely uniform blue figure. The concise region of blue samples indicates correlation between these two torsion angles.

Hydrogen bond details. Hydrogen bond potential (Eq. 3) was kept in our model from our previous work[7]; however, with the introduction of the new vdW_{eff} potential, we re-optimized hydrogen bond parameters to distinguish between base pairing and base stacking interactions. For our model, equilibrium hydrogen bond length $\sigma_{hb,eq}$ is taken to be 2.9Å while $\varepsilon_{hb,max}$ is taken to be 2.0 kcal/mol. Table S2 presents representative potential components for stacking and base pairing interactions taken from PDB ID: 1AL5 structure (A-form dodecamer[8]). Using only the vdW_{eff} potential term, it is observed that base stacking is stronger by ~0.5 kcal/mol over base-pairing interactions. However, with the addition of the hydrogen bond term, base pairing becomes slightly stronger. The GC base pair potential is observed to be 2 kcal/mol more stabilizing than AU base pair largely as a result of hydrogen bonding. This mimics the canonical interactions of the GC basepair with three hydrogen bonds while the AU basepair has two hydrogen bonds. The hydrogen bond term acts on every base pair and is therefore semi-proportional to the number of base pairs. The proportionality helped to increase our predicted folding free energy values to agree better with experiment. Additionally, direct proportionality of the potential energy to base pairs is not desirable since GC, AU, as well as non-canonical basepairs have different stabilities; this leaves room for vdW_{eff} interactions to provide atom type based discrimination.

Table S2. Potential values for representative stacking and canonical base pair structures taken from PDB ID: 1AL5.

	Potential (kcal/mol)		
Structure	vdW_{eff}	Hbond	Total
AU Stack	-2.45	-	-2.45
GC Stack	-3.04	-	-3.04
AU base pair	-2.01	-1.21	-3.22
GC base pair	-2.41	-2.89	-5.30

Directionality and derivative of hydrogen bond potential. Another interesting aspect of the hydrogen bond potential implemented here is that it is directional without including a hydrogen atom, unlike most hydrogen bond potentials [9]. The potential used here accounts for interaction distances and angles of the base pairs by taking the angle between the normal vectors of the bases (see Fig. S16). The analytical force (negative of gradient) of this potential energy, which is needed for energy minimization and molecular dynamics, is given in Fig. S16; notably, the force is dependent on the angle and distance between the bases, with the angle components dependent on positions of all six pseudoatoms.

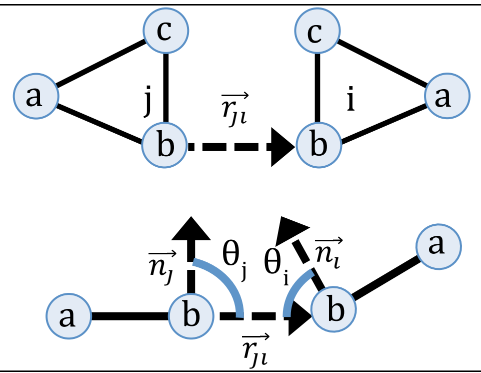

$$\frac{\partial E}{\partial x_{ia}} = -\frac{\varepsilon_{hb,max}}{2}\left[\sin\left(\alpha_k(x_{ia})\right)\left(\frac{\sigma_{hb,eq}}{|\vec{r_{ji}}|}\right)^3\left(\frac{\partial \alpha_k}{\partial x_{ia}}\right)\right]$$

$$\frac{\partial \alpha_k}{\partial x_{ia}} = 2\frac{\partial \theta_i}{\partial x_{ia}} = \frac{-2}{\sqrt{1-\left(\frac{\vec{n_i}\cdot\vec{r_{ji}}}{|\vec{n_i}||\vec{r_{ji}}|}\right)^2}}\frac{\partial\left(\frac{\vec{n_i}\cdot\vec{r_{ji}}}{|\vec{n_i}||\vec{r_{ji}}|}\right)}{\partial x_{ia}}$$

$$\frac{\partial\left(\frac{\vec{n_i}\cdot\vec{r_{ji}}}{|\vec{n_i}||\vec{r_{ji}}|}\right)}{\partial x_{ia}} = \left[\frac{-z_{icb}y_{ji} + y_{icb}z_{ji}}{|\vec{n_i}||\vec{r_{ji}}|} - \frac{(\vec{n_i}\cdot\vec{r_{ji}})\left(-x_{iab}z_{icb} + x_{icb}z_{iab} - z_{icb} + y_{icb}(x_{iab}y_{icb} - x_{icb}y_{iab})\right)}{|\vec{n_i}|^3|\vec{r_{ji}}|}\right]$$

$$\frac{\partial E}{\partial x_{ib}} = -\frac{\varepsilon_{hb,max}}{2}\left[\sin(\alpha_k)\left(\frac{\sigma_{hb,eq}}{|\vec{r_{ji}}|}\right)^3\left(\frac{\partial\alpha_k}{\partial x_{ib}}\right) - 3(1-\cos(\alpha_k))\left(\frac{\sigma_{hb,eq}{}^3}{|\vec{r_{ji}}|^4}\right)\left(\frac{\partial|\vec{r_{ji}}|}{\partial x_{ib}}\right)\right]$$

$$\frac{\partial\alpha_k}{\partial x_{ib}} = 2\frac{\partial\theta_i}{\partial x_{ib}} + 2\frac{\partial\theta_j}{\partial x_{ib}}\ , \qquad \frac{\partial|\vec{r_{ji}}|}{\partial x_{ib}} = \frac{x_{ib} - x_{jb}}{|\vec{r_{ji}}|}$$

$$\frac{\partial\theta_i}{\partial x_{ib}} = \frac{-1}{\sqrt{1 - \left(\frac{\vec{n_i}\cdot\vec{r_{ji}}}{|\vec{n_i}||\vec{r_{ji}}|}\right)^2}}\frac{\partial\left(\frac{\vec{n_i}\cdot\vec{r_{ji}}}{|\vec{n_i}||\vec{r_{ji}}|}\right)}{\partial x_{ib}}, \quad \frac{\partial\theta_j}{\partial x_{ib}} = \frac{-1}{\sqrt{1 - \left(\frac{\vec{n_j}\cdot\vec{r_{ji}}}{|\vec{n_j}||\vec{r_{ji}}|}\right)^2}}\frac{\partial\left(\frac{\vec{n_j}\cdot\vec{r_{ji}}}{|\vec{n_j}||\vec{r_{ji}}|}\right)}{\partial x_{ib}}$$

$$\frac{\partial\left(\frac{\vec{n_i}\cdot\vec{r_{ji}}}{|\vec{n_i}||\vec{r_{ji}}|}\right)}{\partial x_{ib}} = \left[\frac{\left((y_{iab}z_{icb} - y_{icb}z_{iab}) + (z_{icb} - z_{iab})y_{ji} + (y_{iab} - y_{icb})z_{ji}\right)}{|\vec{n_i}||\vec{r_{ji}}|} - \frac{(\vec{n_i}\cdot\vec{r_{ji}})\left((-x_{iab}z_{icb} + x_{icb}z_{iab})(z_{icb} - z_{iab}) + (x_{iab}y_{icb} - x_{icb}y_{iab})(y_{iab} - y_{icb})\right)}{|\vec{n_i}|^3|\vec{r_{ji}}|} - \frac{(\vec{n_i}\cdot\vec{r_{ji}})x_{ji}}{|\vec{n_i}||\vec{r_{ji}}|^3}\right]$$

$$\frac{\partial\left(\frac{\vec{n_j}\cdot\vec{r_{ji}}}{|\vec{n_j}||\vec{r_{ji}}|}\right)}{\partial x_{ib}} = \frac{y_{jab}z_{jcb} - y_{jcb}z_{jab}}{|\vec{n_j}||\vec{r_{ji}}|} - \frac{(\vec{n_j}\cdot\vec{r_{ji}})x_{ji}}{|\vec{n_j}||\vec{r_{ji}}|^3}$$

Fig. S17. Hydrogen bond potential diagram and derivative (negative of force) equations for the *x* coordinate of atoms *a* and *b* on residue *i*. $\vec{n_i}$ and $\vec{n_j}$ are the vectors normal to the plane of residues i and j respectively. $\vec{r_{ji}}$ is the vector between hydrogen bonding atoms from residues j to i (jb and ib in this case). θ_i and θ_j are the angles between the respective normal vectors and vector $\vec{r_{ji}}$. $x_{ia}$is the x-coordinate of atom a on residue i. x_{iab} is the x-coordinate term of the vector from b to a. x_{ji} is the x-coordinate term of the vector $\vec{r_{ji}}$. For

derivatives, atom c follows similarly to atom a.

References

1 WHAM: The Weighted Histogram Analysis Method v. 2.0.9 (http://membrane.urmc.rochester.edu/content/wham).

2 Malmberg, C. G. & Maryott, A. A. DIELECTRIC CONSTANT OF WATER FROM 0-DEGREES-C TO 100-DEGREES-C. *J. Res. Natl. Bur. Stand.* **56**, 1-8, doi:10.6028/jres.056.001 (1956).

3 Gilson, M. K. & Honig, B. H. THE DIELECTRIC-CONSTANT OF A FOLDED PROTEIN. *Biopolymers* **25**, 2097-2119, doi:10.1002/bip.360251106 (1986).

4 Schutz, C. N. & Warshel, A. What are the dielectric "constants" of proteins and how to validate electrostatic models? *Proteins-Structure Function and Bioinformatics* **44**, 400-417, doi:10.1002/prot.1106 (2001).

5 Israelachvili, J. N. in *Intermolecular and Surface Forces (Third Edition)* 291-340 (Academic Press, 2011).

6 Xia, T. B. *et al.* Thermodynamic parameters for an expanded nearest-neighbor model for formation of RNA duplexes with Watson-Crick base pairs. *Biochemistry* **37**, 14719-14735, doi:10.1021/bi9809425 (1998).

7 Xia, Z., Bell, D. R., Shi, Y. & Ren, P. RNA 3D Structure Prediction by Using a Coarse-Grained Model and Experimental Data. *The Journal of Physical Chemistry B* **117**, 3135-3144, doi:10.1021/jp400751w (2013).

8 Conte, M. R., Conn, G. L., Brown, T. & Lane, A. N. Conformational properties and thermodynamics of the RNA duplex r(CGCAAAUUUGCG)2: comparison with the DNA analogue d(CGCAAATTTGCG)2. *Nucleic Acids Research* **25**, 2627-2634, doi:10.1093/nar/25.13.2627 (1997).

9 Lii, J.-H. & Allinger, N. L. Directional hydrogen bonding in the MM3 force field: II. *J. Comput. Chem.* **19**, 1001-1016, doi:10.1002/(SICI)1096-987X(19980715)19:9<1001::AID-JCC2>3.0.CO;2-U (1998).
